# N- and *O*-glycosylation Analysis of Human C1-inhibitor Reveals Extensive Mucin-type *O*-Glycosylation*[Fn FN2]

**DOI:** 10.1074/mcp.RA117.000240

**Published:** 2017-12-12

**Authors:** Kathrin Stavenhagen, H. Mehmet Kayili, Stephanie Holst, Carolien A. M. Koeleman, Ruchira Engel, Diana Wouters, Sacha Zeerleder, Bekir Salih, Manfred Wuhrer

**Affiliations:** From the ‡Center for Proteomics and Metabolomics, Leiden University Medical Center, Leiden, The Netherlands;; §Division of BioAnalytical Chemistry, VU University Amsterdam, Amsterdam, The Netherlands;; ¶Department of Chemistry, Çankırı Karatekin University, Çankırı, Turkey;; ‖Department of Nutrition and Dietetics, Karabuk University, Karabuk, Turkey;; **Department of Chemistry, Hacettepe University, Ankara, Turkey;; ‡‡Department of Immunopathology, Sanquin Research and Landsteiner Laboratory of the AMC, Amsterdam, The Netherlands;; §§Department of Hematology, Academic Medical Center, University of Amsterdam, The Netherlands

**Keywords:** Glycoproteomics, Plasma or serum analysis, Post-translational modifications*, Mass Spectrometry, Glycomics, Glycoproteins*, Complement system, O-glycosylation

## Abstract

Human C1-inhibitor (C1-Inh) is a serine protease inhibitor and the major regulator of the contact activation pathway as well as the classical and lectin complement pathways. It is known to be a highly glycosylated plasma glycoprotein. However, both the structural features and biological role of C1-Inh glycosylation are largely unknown. Here, we performed for the first time an in-depth site-specific *N*- and *O*-glycosylation analysis of C1-Inh combining various mass spectrometric approaches, including C18-porous graphitized carbon (PGC)-LC-ESI-QTOF-MS/MS applying stepping-energy collision-induced dissociation (CID) and electron-transfer dissociation (ETD). Various proteases were applied, partly in combination with PNGase F and exoglycosidase treatment, in order to analyze the (glyco)peptides. The analysis revealed an extensively *O*-glycosylated N-terminal region. Five novel and five known *O*-glycosylation sites were identified, carrying mainly core1-type *O*-glycans. In addition, we detected a heavily *O*-glycosylated portion spanning from Thr_82_-Ser_121_ with up to 16 *O*-glycans attached. Likewise, all known six *N*-glycosylation sites were covered and confirmed by this site-specific glycosylation analysis. The glycoforms were in accordance with results on released *N*-glycans by MALDI-TOF/TOF-MS/MS. The comprehensive characterization of C1-Inh glycosylation described in this study will form the basis for further functional studies on the role of these glycan modifications.

Human C1-inhibitor (C1-Inh)^1^ is a serine protease inhibitor (serpin) and the major regulator of the contact activation pathway via inhibition of factor XIIa, kallikrein and factor XIa, as well as the classical and lectin complement pathways via C1s, C1r, and MASP (1). A C1-Inh deficiency, either because of decreased or dysfunctional expression, is associated with hereditary angioedema (HAE), which results in vascular permeability causing tissue swelling (1). For treatment and prophylaxis of HAE human plasma-derived C1-Inh replacement therapy is commonly applied (2). Also other diseases, such as inflammatory diseases, sepsis and endotoxic shock, may be targeted by C1-Inh therapy (1, 3). To meet this need, recombinant C1-Inh formats are currently being developed (4–7).

C1-Inh is considered as one of the most heavily glycosylated proteins in human plasma (1). The protein consists of 478 amino acids and the calculated molecular mass of C1-Inh is ∼53 kDa without glycans, whereas a much higher apparent molecular mass was observed on SDS-PAGE (>80kDa) because of its heavy glycosylation (8, 9). It has been reported that C1-Inh possesses six occupied *N*- and up to 24 *O*-glycosylation sites (8, 10–13). Of the latter ones ten have been identified with their exact location (10, 11, 13). The protein consists of two domains: (1) the C-terminal domain (serpin domain), which carries three of the six *N*-glycosylation sites, provides the inhibition activity of C1-Inh and is similar to other serpins; and (2) the N-terminal domain, which consists of ∼135–142 amino acid residues (∼113–120 amino acids in the mature protein), featuring the remaining three *N*- and all *O*-glycosylation sites (11, 14). Even though protein glycosylation has a large impact on biological processes, protein stability, and protein functions (15, 16), the structural features as well as biological role of C1-Inh glycosylation is still largely unknown.

To address this, we here present a detailed site-specific *N*- and *O*-glycosylation characterization of plasma derived C1-Inh using a panel of mass spectrometric approaches. The C1-Inh glycosylation as studied here will inform further functional studies in order to understand glycan involvement in C1-Inh function. Furthermore, this plasma-derived human C1-Inh glycosylation will serve as a benchmark for evaluating the glycosylation profiles of recombinant C1-Inh.

## MATERIALS AND METHODS

### 

#### 

##### Materials

Ammonium bicarbonate (AmBiC), hydroxybenzotriazole hydrate (HOBt), iodoacetamide (IAA), 2-mercaptoethanol, Nonidet P-40 (NP-40), PBS, formic acid (FA), sDHB (2-hydroxy-5-methoxy-benzoic acid and 2,5-dihydroxybenzoic acid, 1:9) as well as a 50% sodium hydroxide (NaOH) solution were obtained from Sigma-Aldrich (Steinheim, Germany). Sodium hydrogen phosphate dihydrate (Na_2_HPO_4_x2H_2_O), sodium bicarbonate, DTT, ethanol, potassium dihydrogen phosphate, SDS, sodium chloride and TFA were purchased from Merck (Darmstadt, Germany). 1-ethyl-3-(3-dimethylaminopropyl)carbodiimide hydrochloride (EDC) originated from Fluorochem (Hadfield, UK) and HPLC SupraGradient ACN from Biosolve (Valkenswaard, The Netherlands).

Commercial C1-Inh (Cetor®), isolated from pooled cryo- and 4F-depleted plasma, using ion exchange chromatography and PEG precipitation, was obtained from Sanquin, Amsterdam, The Netherlands.

##### In-solution PNGase F Treatment for Glycopeptide Analysis

For samples to be treated with exoglycosidases, de-*N*-glycosylation was achieved using an in-solution Peptide-*N*-glycosidase F (PNGase F; Roche Diagnostics, Mannheim, Germany) approach prior to in-gel digestion with proteolytic enzymes. Ten microliter C1-Inh (1 μg/μl) were first denatured and reduced with a mixture of 1 μl of 5% SDS and 0.4 m DTT by shaking thoroughly for 5 min at 95 °C. Then, 10 μl PNGase F solution (1 U in 2% NP-40/2 x PBS) was added, followed by an overnight incubation at 37 °C. De-*N*-glycosylated C1-Inh was then subjected to SDS-PAGE and in-gel proteolytic digestion as described below.

##### In-gel Proteolytic Digests for Glycopeptide Analysis

In-gel protease digestion (10 μg protein) of C1-Inh (supplemental Fig. S1), was performed as previously described (17) using either 0.15 μg of trypsin (sequencing grade; Promega, Madison, WI), 1 μg of Proteinase K (*Tritirachium album*, Sigma-Aldrich) or 1 μg Pronase (*Streptomyces griseus,* Sigma-Aldrich) in 30 μl of 25 mm AmBiC.

De-*N*-glycosylated glycopeptides were obtained by in-gel PNGase F treatment (details in released *N*-glycan section) followed by in-gel proteolytic digestion, except for glycopeptide samples that were further treated with exoglycosidases (see in-solution PNGase F treatment). After in-gel PNGase F treatment the gel pieces were washed twice for 5 min with 100 μl 25 mm AmBiC before in-gel proteolytic digestion.

##### Exoglycosidase Treatment of Glycopeptides Samples

(Glyco)-peptides were dried in a centrifugal vacuum concentrator and reconstituted by adding 16 μl of water, 2 μl sodium acetate (50 mm, pH 5.5), 1 μl sialidase (5 mU, Glyko sialidase A; Prozyme, Hayward, CA), and 1 μl of galactosidase (5 mU, Glyko beta-galactosidase, Prozyme). The digestions were carried out overnight at 37 °C.

##### C18-PGC-LC-ESI-QTOF-MS/MS

C18-porous graphitized carbon (PGC)-LC-ESI-QTOF-MS/MS analysis was performed as described previously (18) using a maXis HD QTOF mass spectrometer equipped with a CaptiveSpray nanoBooster source (both Bruker Daltonics) coupled to an Ultimate 3000 × 2 dual analytical nanoUPLC system (Thermo Scientific). The LC-MS setup controlled by Hystar 3.2 (Bruker Daltonics) and data analysis was performed using DataAnalysis 4.2 (Bruker Daltonics).

A combined C18-PGC-LC approach was applied to separate Pronase- and Proteinase K- treated (glyco)peptides (18). The two valve nanoUPLC system was used with the following setup: valve 1 was equipped with a C18 precolumn (C18 PepMap 100, 300 μm x 5 mm, 5 μm, 100 Å, Thermo Scientific) and analytical column (Acclaim PepMap RSLC, 75 μm × 15 cm, 2 μm, 100 Å; Thermo Scientific) and valve 2 with a PGC precolumn (in-house made, 100 μm × 15 mm, 3 μm Hypercarb material; Thermo Scientific) and analytical column (in-house made, 50 μm × 150 mm, 3 μm Hypercarb material; Thermo Scientific). During loading, both precolumns were switched in-line allowing the sample first to pass the C18 precolumn and then to directly load the flow-through, with all unbound compounds, onto the PGC precolumn. In a second step the valves were switched for sequential elution of the compounds from the two precolumns over their corresponding analytical columns. A post-column nano valve directed the flow of the C18 or PGC column system subsequently to the mass spectrometer.

Pronase- and Proteinase K-treated glycopeptides were diluted 10 times and 4 μl were loaded onto the precolumns with loading solvent (99% water/1% ACN/0.05% TFA) at a flow rate of 6 μl/min and column oven temperature of 36 °C. The C18-PGC-LC setup was operated with solvent A (water containing 0.1% FA (*v/v*)) and solvent B (80% acetonitrile/20% water containing 0.1% FA (*v/v*)). First, (glyco)peptides from the C18 columns were eluted with a flow rate of 500 nL/min using a linear gradient (t = 5–35 min, c(B) = 1–55%), followed by column washing and reconditioning. After 28 min a post-column nano-valve was switched and the flow from the PGC columns was sent to the MS. The elution of the PGC columns was performed with a linear gradient (t = 22–55 min, c(B) = 1–40%) at a flow rate of 400 nL/min, followed by column washing and reconditioning.

Ionization was enhanced using a nanoBooster (Bruker Daltonics) with acetonitrile-enriched nitrogen at 0.2 bar. The source parameters were set to a dry gas flow of 3 L/min at 150 °C and a capillary voltage of 1200 V. The mass spectrometer was calibrated using ESI-l-low concentration tuning mixture (Agilent Technologies, Santa Clara, CA). MS acquisition was performed within a mass range of *m*/*z* 50 to *m*/*z* 2800 at a spectra rate of 1 Hz. Basic stepping mode was applied for the MS/MS collision energy (80 and 140%) each for 50% of the time. Collision energies were set as follows: For singly charged precursors 45 eV at *m*/*z* 500, 60 eV at *m*/*z* 800, 80 eV at *m*/*z* 1300; for doubly charged precursors 25 eV at *m*/*z* 500, 47 eV at *m*/*z* 800, 60 eV at *m*/*z* 1300, for precursors with three and more charges 20 eV at *m*/*z* 500, 45 eV at *m*/*z* 800, 65 eV at *m*/*z* 1300. MS/MS was performed on the three most abundant precursor ions at a spectra acquisition rate of 0.5 Hz to 2 Hz depending on the precursor intensity (18).

##### C18-RP-LC-ESI-QTOF-MS/MS

C18-RP-LC-ESI-QTOF-MS/MS analysis was performed on the same LC-MS system as used for C18-PGC-LC-ESI-QTOF-MS/MS. All trypsin-treated samples were diluted 30 times with water and 5 μl were loaded onto a C18 precolumn (C18 PepMap 100, 100 μm x 2 cm, 5 μm, 100 Å, Dionex/Thermo Scientific, Breda, The Netherlands) with 15 μl/min of loading solvent (water containing 0.1% ACN/0.1% FA) for 2 min. The analytes were separated on a C18 analytical column (Acclaim PepMap RSLC, 75 μm × 15 cm, 2 μm, 100 Å, Dionex/Thermo Scientific) at 32 °C column oven temperature. Elution was performed at a flow rate of 0.7 μl/min with solvent A (water containing 0.1% formic acid (FA) (v/v)) and solvent B (95% acetonitrile (ACN)/5% water containing 0.1% FA (v/v)). A linear gradient of 3–31.7% solvent B in 25 min was applied followed by column washing and reconditioning.

The MS was operated in stepping-energy CID mode as described previously (setup two in (19)). To acquire data for relative quantitation the MS was operated in MS only mode. For electron-transfer dissociation (ETD) experiments the MS parameters were set as described for stepping-energy CID, except of the collision RF, which was set to 500 and 800 Vpp in basic stepping mode (each 50% of the time). The ICC target was set to 3 Mio for an accumulation time of max. 600 ms. The reagent injection was 45 ms and the extended reaction time 5 ms. ETD precursors were selected using a target list obtained from CID runs.

##### Data Evaluation of LC-MS/MS Spectra

DataAnalysis 4.2 software (Bruker Daltonics) was used to analyze glycopeptides of C1-Inh (P05155) by manually scanning for glycan oxonium ions. The defined stepping-energy CID glycopeptide spectra were analyzed manually to identify the glycan structure and the mass of the peptide backbone as described previously (18), including carbamidomethylation as a fixed and oxidation as a variable modification. Additionally, also lower mass range oxonium ions of stepping-energy CID spectra were used to characterize the glycan portion. The ratio of HexNAc fragments in higher-energy CID can be a marker for the presence of GlcNAc in the glycopeptide (20). A high ratio of *m*/*z* 138 ([HexNAc-CH_6_O_3_]^+^) + *m*/*z* 168 ([HexNAc-2H_2_O]^+^) relative to *m*/*z* 126 ([HexNAc-C_2_H_6_O_3_]^+^) + *m*/*z* 144 ([HexNAc-C_2_H_4_O_2_]^+^) is diagnostic for a GlcNAc-containing glycopeptide, close to equal intensities of *m*/*z* 138 + *m*/*z* 168 compared with *m*/*z* 126 + *m*/*z* 144 are indicative for only GalNAc-containing glycopeptides.

For a selected list of identified glycopeptides with known compositions and retention times, based on CID spectra, also ETD spectra were manually analyzed, allowing a mass deviation of 10 ppm (QTOF) and 0.1 Da (IT).

For relative quantitation signal intensities of all tryptic glycopeptides and partially also of miss-cleaved ones (up to one miss cleavage) were extracted in an automated manner using LaCy tools (version 1.0.0) (21). LaCy tools settings were as follows: sum spectrum resolution = 100; mass window 0.07 Th; time window 18 s; minimum percentage of the total theoretical isotopic distribution = 95%, background window = 10 Th. The analyte was included for relative quantitation based on the following criteria: signal-to-noise of at least 9; average mass error of ±10 ppm, average isotopic pattern quality (IPQ) score ≤0.25. The samples were analyzed in triplicates. The data was normalized based on the total intensity of all compounds and the standard deviation was calculated.

##### Released N-glycan Analysis

In-gel *N*-glycan release (10 μg protein) was performed as previously described (17) with minor modifications. Different from the protocol, the gel bands were washed with 25 mm sodium bicarbonate, pH 8, instead of AmBiC. For the *N*-glycan release, 20 μl to 30 μl PNGase F solution (2 U (Roche Diagnostics) in 2% Nonidet P-40 (NP-40) and 2.5xPBS) were used.

Released *N*-glycans were derivatized by ethyl esterification (22) followed by glycan purification by hydrophilic interaction chromatography (HILIC)-solid phase extraction (SPE) using cotton thread modified from a protocol described previously (23). The *N*-glycans were eluted in 10 μl water. From this, 5 μl were used for mass spectrometric analysis by spotting them onto an anchor chip matrix-assisted laser desorption dissociation (MALDI) target plate (Bruker Daltonics, Bremen, Germany) and cocrystallized with 1 μl of 5 mg/ml sDHB (2-hydroxy-5-methoxy-benzoic acid and 2,5-dihydroxybenzoic acid, 1:9, Sigma-Aldrich) in 50% ACN/50% water containing 1 mm NaOH. MALDI-TOF-MS spectra were acquired using an UltrafleXtreme mass spectrometer (Bruker Daltonics) in positive ion reflector mode. Spectra were obtained over a mass window of *m*/*z* 1000 to *m*/*z* 5000 with suppression up to *m*/*z* 900 for a total of 20,000 shots. Tandem mass spectrometry (MALDI-TOF/TOF-MS/MS) was performed for structural elucidation via fragmentation in gas-off TOF/TOF mode.

A compound list of C1-Inh *N*-glycans was manually curated and relative quantitation of the *N*-glycoforms was performed using an in-house developed software for automated data processing MassyTools 1.0 (24). Only glycan compositions that have been confirmed by MS/MS and their directly related compositions (± one monosaccharide) were taken into account for relative quantitation. Detailed information of the released *N*-glycan sample preparation and analysis is provided in the supplemental data.

##### Experimental Design and Statistical Rationale

A detailed *N*- and *O*-glycosylation analysis of C1-Inh was performed using various MS-based approaches. C1-Inh glycopeptides were generated by subjecting the purified glycoprotein to various protease treatments with and without the addition of PNGase F and exoglycosidases for *N*-glycan release and glycan trimming, respectively. This resulted in *N*-, *O*- and *N*-/*O*-glycopeptides of different complexity to achieve a high glycopeptide coverage. Additionally, C1-Inh *N*-glycans were released and analyzed. Sample preparation, followed by MS analysis was performed in triplicates (glycopeptide analysis) or quadruplicates (*N*-glycan analysis) and the average relative distribution of glycopeptides, *N*-glycans and their respective standard deviations were calculated.

## RESULTS

### 

#### 

##### Site-specific O-glycosylation Analysis of C1-Inh

For *O*-glycosylation site identification and characterization C1-Inh was subjected to in-gel trypsin, Pronase, and Proteinase K treatment. The latter two broad-specificity proteases commonly result in smaller peptide portions to reduce sample complexity for tandem MS analysis. To further decrease sample heterogeneity and enhance *O*-glycosylation site identification a portion of these digests were de-*N*-glycosylated by PNGase F and partially also processed with exoglycosidases such as sialidase and galactosidase. This approach aimed to trim down short mucin-type *O*-glycans, to obtain *O*-glycopeptides with a single *N*-acetylhexosamine (HexNAc) or HexNAc-hexose (Hex) moiety attached to the *O*-glycosylation site, which allowed a more reliable site-specific analysis. Samples were analyzed by nanoLC-MS/MS analysis applying different tandem MS modes to obtain more structural information of the glycopeptides. For this, we applied a C18-PGC-LC-ESI-QTOF-MS/MS approach that has recently been developed by us and facilitates higher glycopeptide coverage after Pronase and Proteinase K treatment (18). Next to the targeted ETD mode, stepping-energy CID was applied for glycopeptide assignment, resulting in b- and y-type peptide fragments, in addition to B- and Y-type glycan-derived fragments (18, 19). A detailed description of the individual *O*-glycosylation sites is given in the following and a list with all identified *O*-glycopeptides is provided in supplemental Table S1.

**Fig. 1.**
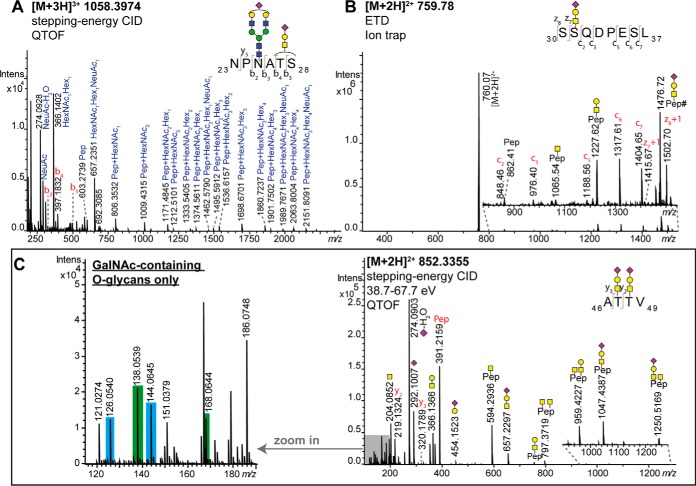
**Mass spectrometric elucidation of C1-Inh *O*-glycosylation.** Representative *O*-glycopeptide mass spectra are shown. *A*, Stepping-energy CID spectrum of a Pronase-treated glycopeptide with the peptide sequence _23_NPNATS_28_ carrying a monosialylated core 1 structure and a monosialylated diantennary *N*-glycan, confirming the *N*-glycosylation site Asn25 and indicating either Thr27 or Ser28 to be *O*-glycosylated; *B*, The Pronase-generated glycopeptide _30_SSQDPESL_37_ with a monosialylated core 1 glycan attached, confirming the *O*-glycosylation site Ser31. The sample was analyzed by ion trap-MS/MS using ETD (experimental details in supplemental data). # indicates the loss of an acetyl group; *C*, Stepping-energy CID spectrum of a Pronase-treated glycopeptide with the peptide sequence _46_ATTV_49_ with two monosialylated core 1 structures attached, confirming the *O*-glycosylation sites Thr47 and Thr48. The zoom-in shows the lower mass oxonium ions. Close to equal intensities of *m/z* 138 ([HexNAc-CH_6_O_3_]^+^) + *m/z* 168 ([HexNAc-2H_2_O]^+^) compared with *m/z* 126 ([HexNAc-C_2_H_6_O_3_]^+^) + *m/z* 144 ([HexNAc-C_2_H_4_O_2_]^+^) are diagnostic for an only GalNAc-containing glycan portion attached to the peptide (20). Glycan depiction: blue square = N-acetylglucosamine, yellow square = N-acetylgalactosamine, yellow circle = galactose, green circle = mannose, red triangle = fucose, purple diamond = N-acetylneuraminic acid.

The first *O*-glycosylation site within the protein sequence of C1-Inh is either Thr27 or Ser28 identified within the peptide portion _23_NPNATS_28_ carrying an *N*-glycan (HexNAc_4_Hex_5_NeuAc_1_) and a monosialylated core 1 *O*-glycan (HexNAc_1_Hex_1_NeuAc_1_) (Fig. 1*A*). The peptide and glycan portions were identified by the exact mass and several b- and y-ions, as well as B- and Y-ions. Diagnostic ions, such as *m*/*z* 1462.5790 [M+H]^+^ (peptide + HexNAc_2_Hex_1_NeuAc_1_) revealed the *O*-glycan nature next to the *N*-glycan portion, however, the exact location could not be unambiguously identified.

The *O*-glycosylation site Ser31 was identified by applying ETD to the glycopeptide _30_SSQDPESL_37_ with a monosialylated core 1 *O*-glycan attached (Fig. 1*B*). The fragment ions z_8_ and z_7_ with the *O*-glycan attached were observed next to c_2_ and c_3_ peptide + glycan fragments identifying Ser31 as glycosylation site.

**Fig. 3.**
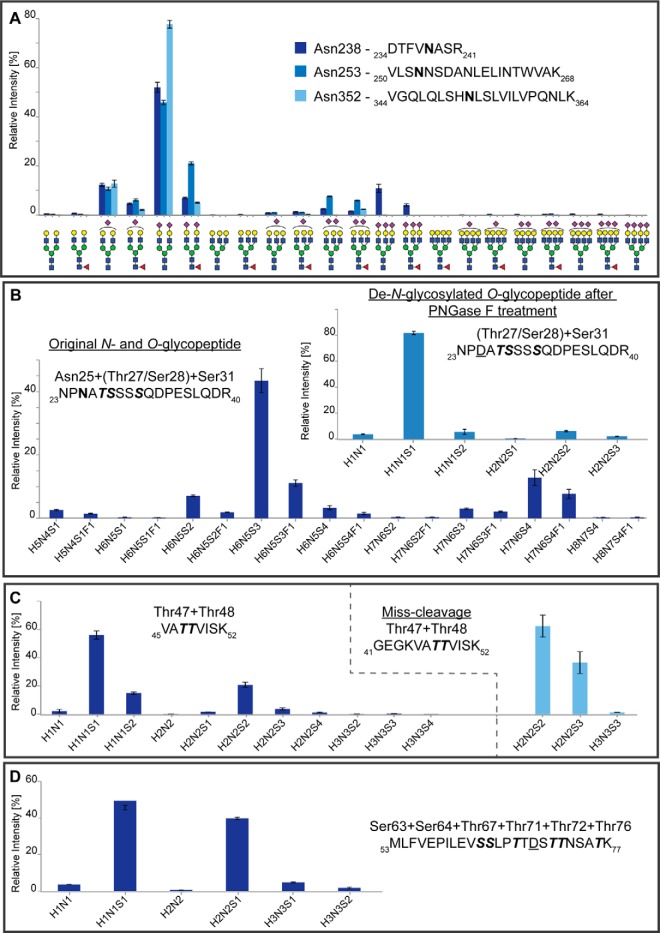
**Site-specific relative quantification of C1-Inh *N*- and *O*-glycoforms.** The signal intensities were normalized by the sum of all glycopeptides at each site containing the same peptide moiety. The average intensities and standard deviations of the glycoforms were calculated based on the analysis of triplicates. *A*, Relative distribution of the tryptic *N*-glycopeptides of Asn238, Asn253 and Asn352. *B*, Relative distribution of the tryptic glycopeptide _23_NPNATSSSSQDPESLQDR_40_ containing *N*-glycans attached to Asn25 and *O*-glycans attached to Thr27/Ser28 and/or Ser31. The insert diagram contains the *O*-glycopeptide distribution after enzymatic de-*N*-glycosylation using PNGase F. *C*, Relative distribution of the tryptic and miss-cleaved *O*-glycopeptides covering the sites Thr47, Thr48. *D*, Relative distribution of the tryptic *O*-glycopeptides covering the sites Ser63, Ser64, Thr67, Thr71, Thr72, Thr76. (*D* indicated deamidation after PNGase F treatment.)

The analysis of tryptic de-*N*-glycosylated *O*-glycopeptides revealed the presence of non-, mono- and disialylated core 1 *O*-glycans attached to the peptide portion _23_NP*D*ATSSSSQDPESLQDR_40_ (*D* indicating deamidation after PNGase F treatment) covering Thr27/Ser28 and Ser31 (supplemental Table S1*B*). Lower stepping-energy CID was applied in order to obtain more intense oxonium ions for glycan structural assignment (supplemental Fig. S2*B*, S2*C*, S2*D*), and the stepping-energy CID spectrum was used to confirm the peptide portion (supplemental Fig. S2*A*). Relative quantitation of the de-*N*-glycosylated *O*-glycopeptides containing the sites Thr27/Ser28 and Ser31 shows that around 82% of the glycopeptides contain one monosialylated core 1 *O*-glycan attached to the peptide at a time (Fig. 3*B*, inset, supplemental Table S4).

The glycosylation sites Thr47 and Thr48 were identified based on the Pronase-treated glycopeptide _46_ATTV_49_ with two monosialylated core 1 structures attached (Fig. 1*C*). The peptide and glycan portions were identified by the exact mass and several b- and y-ions, as well as B- and Y-ions. During this study also lower mass range oxonium ions of stepping-energy CID spectra were taken into account to identify the glycan portion. As reported by Halim *et al.* the ratio of HexNAc fragments in higher-energy CID can be a marker for the presence of GlcNAc in the glycopeptide (20). A high ratio of *m*/*z* 138 ([HexNAc-CH_6_O_3_]^+^) + *m*/*z* 168 ([HexNAc-2H_2_O]^+^) relative to *m*/*z* 126 ([HexNAc-C_2_H_6_O_3_]^+^) + *m*/*z* 144 ([HexNAc-C_2_H_4_O_2_]^+^) is diagnostic for a GlcNAc-containing glycopeptide and close to equal intensities of *m*/*z* 138 + *m*/*z* 168 compared with *m*/*z* 126 + *m*/*z* 144 are indicative for only GalNAc-containing glycopeptides. A representative example of an *O*-glycopeptide (GalNAc only) and the same glycopeptide with an additional *N*-glycan attached is given in supplemental Fig. S3. The presence of four GlcNAc residues in the *N*-glycan portion clearly results in elevated relative signals for *m*/*z* 138 + *m*/*z* 168 compared with *m*/*z* 126 + *m*/*z* 144. Based on close to equal intensities of these signals for the glycopeptide _46_ATTV_49_ (Fig. 1*C*) it can be concluded that GalNAc is prevailing and consequently two monosialylated core 1 structures are attached to the peptide portion. To further study the glycan microheterogeneity of these glycosylation sites the tryptic *O*-glycopeptides with the peptide portion _45_VATTVISK_52_ were analyzed by lower stepping-energy CID (supplemental Fig. S4*D*, S4*E* and S5*E*, S5*F*, S5*G*). Compared with the stepping-energy CID spectrum (supplemental Fig. S4*C*) the lower stepping-energy CID spectrum (supplemental Fig. S4*D*) contains more intense oxonium ions for glycan structural elucidation. Using this approach it was possible to identify non-, mono- and disialylated core 1, as well as a monosialylated core 2 structure attached to the glycosylation sites Thr47/Thr48 (supplemental Fig. S4*D*, S4*E* and S5*E*, S5*F*, S5*G*). Based on the lower mass range oxonium ion pattern it can be further verified that the glycosylation sites Thr47/Thr48 contain mainly core 1 *O*-glycans (supplemental Fig. S4*B* and S5A, S5*B*, S5*C*) and partially also core 2 *O*-glycans (supplemental Fig. S4*A*). Relative quantitation of tryptic *O*-glycopeptides with the peptide portion _45_VATTVISK_52_ revealed approx. 73% of the glycopeptides to be occupied by core 1 *O*-glycans (Fig. 3*C*, supplemental Table S4). However, the missed cleaved peptide species _41_GEGKVATTVISK_52_ contains almost exclusively glycopeptides with the composition HexNAc_2_Hex_2_NeuAc_2_ and HexNAc_2_Hex_2_NeuAc_3_, indicating a bias in trypsin cleavage based on the *O*-glycan portion. With both peptide variants the glycan compositions containing HexNAc_3_Hex_3_NeuAc_2–4_ indicative of a core 2 *O*-glycan are <1.1% (Fig. 3*C*, supplemental Table S4).

**Fig. 2.**
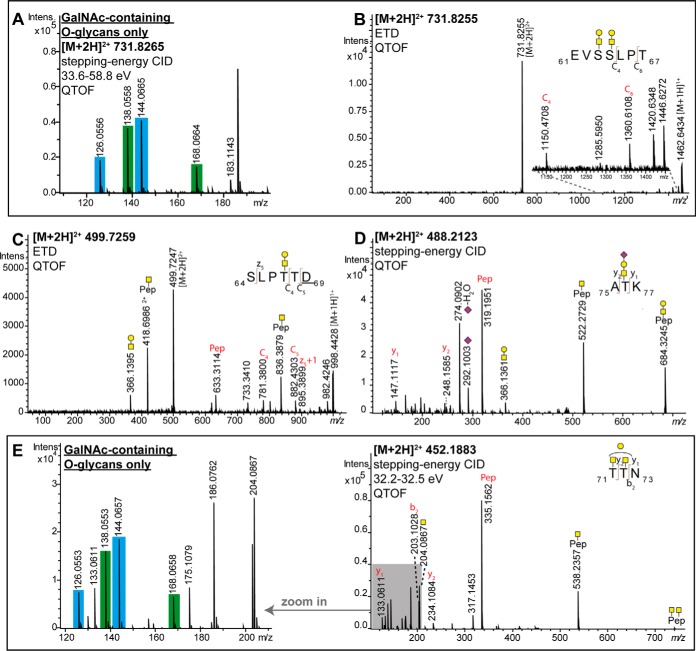
**Mass spectrometric elucidation of C1-Inh *O*-glycosylation.** Representative *O*-glycopeptide mass spectra are shown. *A*, Lower mass oxonium ion region of a stepping-energy CID spectrum of the glycopeptide _61_EVSSLPT_67_ carrying two HexNAc_1_Hex_1_ structures. Almost equal intensities of *m/z* 138 + *m/z* 168 compared with *m/z* 126 + *m/z* 144 are diagnostic for an only GalNAc-containing glycan portion attached to the peptide, confirming the presence of two core 1 O-glycans; *B*, A Proteinase K-treated glycopeptide after exoglycosidase treatment with the peptide sequence _61_EVSSLPT_67_ carrying two HexNAc_1_Hex_1_ structures, confirming the *O*-glycosylation sites Ser63 and Ser64. The sample was analyzed by QTOF-MS/MS using ETD. *C*, The Proteinase K-treated glycopeptide after PNGase F and exoglycosidase treatment with the peptide sequence _64_SLPTT*D*_69_ (*D* indicated deamidation after PNGase F treatment) and a HexNAc_1_Hex_1_ *O*-glycan attached, confirming the *O*-glycosylation site Thr67. The sample was analyzed by QTOF-MS/MS using ETD. *D*, Stepping-energy CID spectrum of a Pronase-treated glycopeptide with the peptide sequence _75_ATK_77_ carrying a monosialylated core 1 structure, confirming the *O*-glycosylation site Thr76; *E*, Stepping-energy CID spectra of a Pronase-treated glycopeptide after exoglycosidase treatment with the peptide sequence _71_TTN_73_ carrying two monosialylated core 1 structure, confirming the O-glycosylation site Thr71 and Thr72. The zoom in shows the lower mass oxonium ions. An almost equal ratio of *m/z* 138 + *m/z* 168 compared with *m/z* 126 + *m/z* 144 is diagnostic for an only GalNAc-containing glycan portion attached to the peptide, confirming the presence of two core 1 O-glycans.

The glycosylation sites Ser63 and Ser64 were identified based on the ETD spectrum of the Pronase- and exoglycosidases-treated glycopeptide _61_EVSSLPT_67_ carrying two non-sialylated core 1 *O*-glycans (Fig. 2*B*). The diagnostic ions c_4_ and c_6_ exclude Thr67 as glycosylation site and confirm the occupation of Ser63 and Ser64. The stepping-energy CID spectrum and lower stepping-energy CID spectrum of this glycopeptide provided further evidence that the glycopeptide contains two individual core 1 *O*-glycans instead of one core 2 *O*-glycan (supplemental Fig. S6). The stepping-energy CID spectrum features lower mass range oxonium ions that indicate only GalNAc-containing *O*-glycans (approximately equal signal intensities for *m*/*z* 138 ([HexNAc-CH_6_O_3_]^+^) + *m*/*z* 168 ([HexNAc-2H_2_O]^+^) compared with *m*/*z* 126 ([HexNAc-C_2_H_6_O_3_]^+^) + *m*/*z* 144 ([HexNAc-C_2_H_4_O_2_]^+^) (Fig. 2*A*). Accordingly, both stepping-energy CID and lower stepping-energy CID spectra contained no diagnostic oxonium ions that would indicate core 2 *O*-glycans such as HexNAc_2_ or HexNAc_2_Hex_1_.

The *O*-glycosylation site Thr67 was identified from the ETD spectrum of the Proteinase K- and exoglycosidases-treated glycopeptide _64_SLPTT*D*_69_ (*D* indicates the deamidated Asn caused by PNGase F treatment) with a nonsialylated core 1 *O*-glycan (Fig. 2*C*). The fragment ions c_4_ and z_5_ identify the *O*-glycosylation site Thr67.

The *O*-glycosylation sites Thr71 and Thr72 were identified by Pronase- and exoglycosidases-treated glycopeptides. The glycopeptide _71_TTN_73_ at *m*/*z* 452.1883 [M+2H]^2+^ was observed with a single HexNAc together with a HexNAc_1_Hex_1_, because of incomplete galactosidase digestions (Fig. 2*E*). The peptide sequence could be confirmed via the exact mass of the peptide ion at *m/z* 335.1562 [M+H]^+^ as well as peptide fragment ions. This peptide sequence is, however, not unique and could also result from _67_TTN_69_. Similarly, glycopeptides with the peptide sequences TTNS were found, which could as well correspond to the peptides _71_TTNS_74_ or _67_TTNS_70_. Ultimately, the same sample also contained a glycopeptide with the unique peptide sequence _71_TTNSAT_76_ confirming the glycosylation sites Thr71 and Thr72, because no further cleavage products were found to support the other sequence (supplemental Table S1*C*). The lower mass range oxonium ions of these glycopeptides featured close to equal signal intensities for *m*/*z* 138 ([HexNAc-CH_6_O_3_]^+^) + *m*/*z* 168 ([HexNAc-2H_2_O]^+^) compared with *m*/*z* 126 ([HexNAc-C_2_H_6_O_3_]^+^) + *m*/*z* 144 ([HexNAc-C_2_H_4_O_2_]^+^), indicating the presence of two GalNAc-containing core 1 *O*-glycans only instead of one core 2 *O*-glycan (Fig. 2*E*, supplemental Fig. S7).

The *O*-glycosylation site Thr76 was identified from a Pronase-treated glycopeptide _75_ATK_77_, carrying a monosialylated core 1 structure. The peptide and glycan portion were identified by the exact mass and several b- and y-ions as well as B- and Y-ions (Fig. 2*D*).

The de-*N*-glycosylated tryptic *O*-glycopeptides with the peptide sequence _53_MLFVEPILEVSSLPTT*D*STTNSATK_77_ (*D* indicates deamidation after PNGase F treatment) were analyzed to determine the glycan microheterogeneity of this region, containing the glycosylation sites Ser63, Ser64, Thr 67, Thr71, Thr72, Thr76. The observed fragmentation spectra indicated two simultaneously occupied glycosylation sites (supplemental Fig. S8), featuring mono- and non-sialylated core 1 *O*-glycans. Relative quantitation revealed that approx. 50% of the glycopeptides contain one monosialylated core 1 *O*-glycan and 40% one monosialylated core 1 and one non-sialylated core 1 *O*-glycan (Fig. 3*D*, supplemental Table S4).

**Fig. 4.**
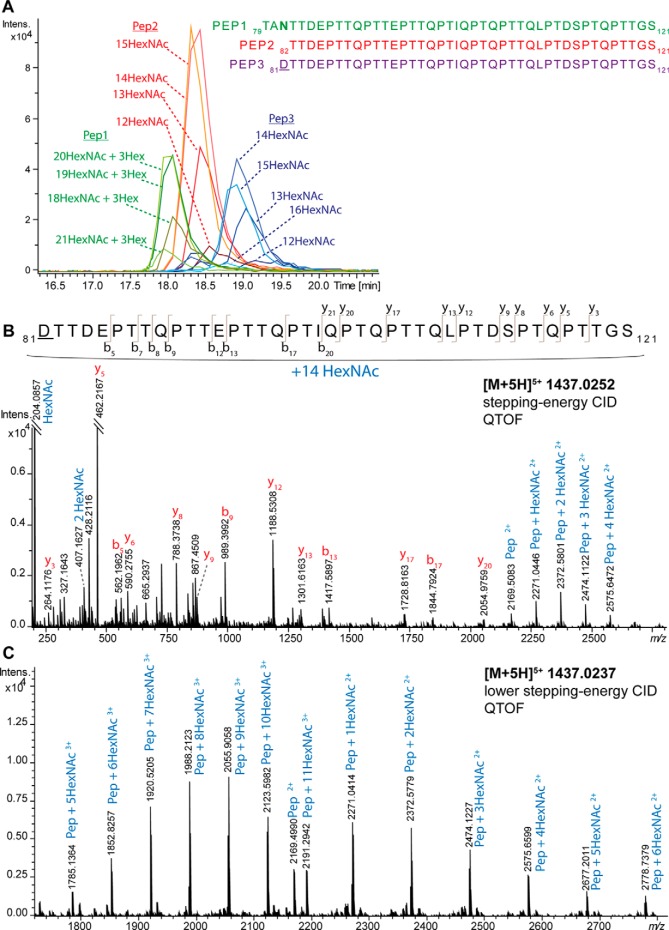
**C18-PGC-LC-ESI-QTOF-MS/MS analysis of Pronase-generated *O*-glycopeptides with multiple glycosylation sites after PNGase F *N*-glycan release and exoglycosidase treatment with sialidase and galactosidase.** *A*, Extracted ion chromatograms of three different glycopeptide clusters spanning from Thr79 to Ser121 with different glycan compositions attached. Pep1 = _79_TANTTDEPTTQPTTEPTTQPTIQPTQPTTQLPTDSPTQPTTGS_121_; Pep2 = _82_TTDEPTTQPTTEPTTQPTIQPTQPTTQLPTDSPTQPTTGS_121_; Pep3 = _81_*D*TTDEPTTQPTTEPTTQPTIQPTQPTTQLPTDSPTQPTTGS_121_ (*D* indicates the deamidated Asn caused by PNGase F treatment) *B*, Stepping-energy CID spectrum of Pep3 with 14 HexNAc residues, indicating up to 14 occupied *O*-glycosylation sites. *C*, Stepping-energy CID spectrum of the same glycopeptide as in panel B using lower-energy stepping-energy CID (stepping energy is set to 60 and 80% each half of the time (instead of 80–140%) with a focus on the glycan-derived Y-ions.

The analysis of Pronase-treated samples after *N*-glycan release and subsequent glycan trimming with exoglycosidases also revealed a highly *O*-glycosylated peptide region with up to 16 occupied *O*-glycosylation sites. Using C18-PGC-LC-ESI-QTOF-MS/MS analysis, three different glycopeptide clusters eluted close to each other featuring three peptide portions (Fig. 4*A*). Two of them only contained occupied *O*-glycosylation sites, Thr82-Ser121 and Asp81-Ser121. For the latter one, Asp81 was identified as deamidated Asn because of PNGase F treatment. The two peptides with only occupied *O*-glycosylation sites contained 18 potential *O*-glycosylation sites of which up to 16 were found to be occupied (Fig. 4*A*). The glycopeptide at *m/z* 1437.0252 [M+5H]^5+^ covered 14 HexNAc residues (_81_*D*TTDEPTTQPTTEPTTQPTIQPTQPTTQLPTDSPTQPTTGS_121_; *D* indicates the deamidated Asn caused by PNGase F treatment) (Fig. 4*B*). The peptide mass (*m*/*z* 2169.5083 [M+2H]^2+^) was determined from the tandem MS spectrum and the sequence was confirmed by several b- and y- ions (Fig. 4*B*). A lower-stepping-energy CID fragmentation spectrum with a zoom-in of the peptide + HexNAc fragments of this highly glycosylated peptide further proved the glycopeptide identity (Fig. 4*C*). Because the glycopeptide spectrum also contains a lower intensity signal at *m*/*z* 407.1627 indicative for HexNAc_2_, it might be possible that a small portion of these glycopeptides also contains core 2 *O*-glycans. Evaluating the low mass range oxonium ions of the different *O*-glycopeptides and combined *N*-/*O*-glycopeptides it can be seen that the *N*-glycan- and thus GlcNAc-containing *N*-/*O*-glycopeptide shows slightly higher signal intensities for *m*/*z* 138 ([HexNAc-CH_6_O_3_]^+^) + *m*/*z* 168 ([HexNAc-2H_2_O]^+^) compared with *m*/*z* 126 ([HexNAc-C_2_H_6_O_3_]^+^) + *m*/*z* 144 ([HexNAc-C_2_H_4_O_2_]^+^) (supplemental Fig. S9*C*). In contrast the corresponding *O*-glycopeptides show close to equal ratios of *m*/*z* 138 + *m*/*z* 168 compared with *m*/*z* 126 + *m*/*z* 144, indicating mainly GalNAc-containing *O*-glycopeptides (supplemental Fig. S9*A*, S9*B*). Thus, we conclude that the up to 16 present HexNAc residues attached to the glycan portion _81_*D*TTDEPTTQPTTEPTTQPTIQPTQPTTQLPTDSPTQPTTGS_121_ (*D* indicates the deamidated Asn caused by PNGase F treatment) represent up to 16 occupied *O*-glycosylation sites, with possible smaller amounts of core 2 *O*-glycans.

**Fig. 5.**
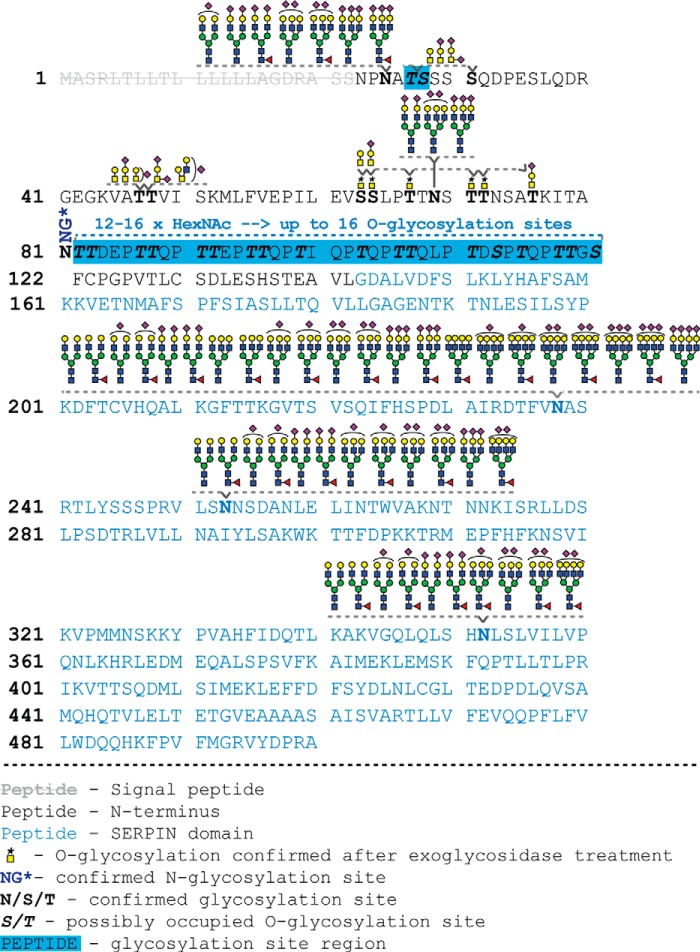
**A schematic representation of the *N*- and *O*-glycosylation of C1-Inh with the serpin (blue letters) and N-terminal domain (black letters).** All determined glycosylation sites are in bold letters depicted with their corresponding glycoforms. *O*- and *N*-glycosylation sites that have been identified after exoglycosidase treatment are marked with a *, because their microheterogeneity was not detected. Possible *O*-glycosylation sites that were not identified with their exact location are indicated in bold and italic letters.

In total, 26 *O*-glycosylation sites, carrying mainly core 1 *O*-glycans, were identified on the N-terminal domain of human C1-Inh (Fig. 5). Nine of them (Ser31, Thr47, Thr48, Ser63, Ser64, Thr67, Thr71, Thr72, and Thr76) were detected with their exact location. Additionally, one *O*-glycosylation site was identified as either Thr27 or Ser28, next to a heavily glycosylated mucin-type *O*-glycan region spanning from Thr82 to Ser121 with up to 16 *O*-glycosylation sites.

##### Released N-glycan Analysis of C1-Inh

**Fig. 6.**
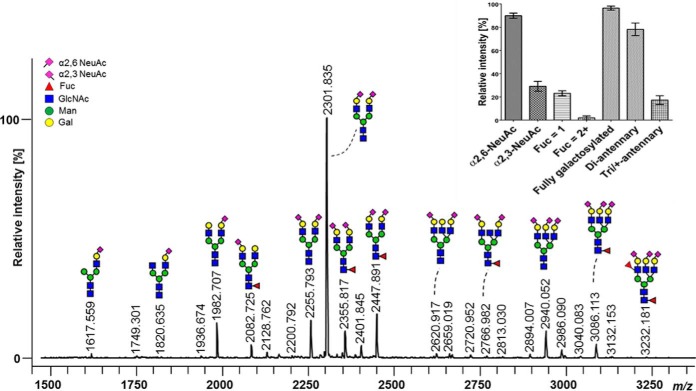
**Total released *N*-glycan profiling of human C1-Inh.** MALDI-TOF-MS analysis of C1-Inh *N*-glycans was performed after PNGase F release and linkage-specific sialic acid derivatization. Glycans were assigned based on MS/MS data and literature. *N*-acetylneuraminic acid (NeuAc) in α2,3- and α2,6-linkage are represented by a purple diamond pointing to the left and right, respectively. The insert shows the relative quantification of *N*-glycan features from quadruplets including antenna-fucosylation (defined as amount of fucoses equal or higher than two) and antennarity.

To get an overview of the overall C1-Inh *N*-glycosylation on all six sites, released *N*-glycans were analyzed using linkage-specific sialic acid derivatization and MALDI-TOF-MS analysis (Fig. 6, supplemental Table S2). The majority of the *N*-glycan structures were sialylated species, among which a diantennary, α2,6-linked disialylated *N*-glycan was the most abundant one (Hex_5_HexNAc_4_(α2,6)N-acetylneuraminic acid (NeuAc_2_)) (supplemental Fig. S10).

Approximately 90% of C1-Inh *N*-glycans contained at least one α2,6-linked NeuAc and about 30% featured α2,3-linked NeuAc. Approximately 25% of the *N*-glycans were monofucosylated and MS/MS analysis identified fucoses to be mainly core-linked. Multi-fucosylation (two or more fucoses), indicative of antenna fucosylation, was less than 5%. C1-Inh *N*-glycans were almost completely galactosylated (95%) and most of the structures were diantennary glycans (80%), next to tri- or tetraantennary glycans (below 20%).

##### Site-specific N-glycopeptide Analysis of C1-Inh

In order to identify *N*-glycosylation sites and site-specific glycan microheterogeneity, proteolytic treatment was carried out using trypsin, Proteinase K and Pronase. The samples were analyzed by C18-RP-LC-ESI-QTOF-MS/MS and C18-PGC-LC-ESI-QTOF-MS/MS using stepping-energy CID. All six potential *N*-glycosylation sites of C1-Inh were identified and are listed in supplemental Table S3 and representative tandem MS spectra of each site are shown in Fig. 1*C* and supplemental Fig. S11, S12. The corresponding glycoforms of each *N*-glycosylation site are depicted in Fig. 5 and are elaborated in more detail in the following.

Trypsin digestion of C1-Inh allowed identification and characterization of glycosylation sites Asn238, Asn253, and Asn352. Glycopeptides were identified based on their tandem MS spectra. Additional glycopeptides from these sites with glycoforms that follow the same biosynthetic pathway were identified by their exact mass on MS level. The relative abundances of the different glycoforms attached to the corresponding tryptic glycopeptides were extracted in an automated manner using the software package LaCytools (21). The relative intensities for each glycosylation site were normalized to the total intensity of all glycoforms attached to the same peptide moiety. (Fig. 3*A*, supplemental Table S4).

For Asn238, 21 *N*-glycoforms were quantified, with HexNAc_4_Hex_5_NeuAc_2_ (52.0%) being the most abundant one. The majority of the *N*-glycans are diantennary (77%), followed by triantennary (21.4%) and tetraantennary ones (1.6%). While the glycans are almost completely sialylated (98.6%) only 20.1% are fucosylated.

The relative quantitation of 12 glycoforms of the glycosylation site Asn253 identified HexNAc_4_Hex_5_NeuAc_2_ (45.8%) as the most abundant structure. Around 84.1% of the *N*-glycans were diantennary, next to another 15.5% triantennary and less than 1% tetraantennary ones. Asn253 features almost complete sialylation and ∼34.7% fucosylation.

For Asn352, six *N*-glycoforms were used for relative quantitation. Next to four diantennary sialylated *N*-glycans also two triantennary sialylated *N*-glycan were quantified. Again, HexNAc_4_Hex_5_NeuAc_2_ was the most abundant structure with 77.7%. Four of these *N*-glycans were fucosylated representing 9.6%.

The glycoforms present on Asn25 were identified by Pronase-treated glycopeptides. Pronase digestion was used for *N*-glycosylation site characterization to generate smaller peptide portions and thereby overcoming interference from the two *O*-glycosylation sites near (Ser31 and Thr27/Ser28) to Asn25. Site-specific elucidation of the *N*-glycoforms was facilitated by tandem mass spectrometry, which allowed the distinction between *O*-glycan and *N*-glycan on this peptide moiety. Asn25 features di- and triantennary *N*-glycans with and without core fucosylation ranging from mono- to trisialylation. For relative quantitation, 18 different glycan compositions were detected attached to the tryptic glycopeptides around Asn25 (_23_NPNATSSSSQDPESLQDR_40_) (Fig. 3*B*). To identify the contribution of the *O*-glycans, the de-*N*-glycosylated species of this peptide were analyzed after PNGase F treatment as discussed in the *O*-glycosylation analysis section (Fig. 3*B* insert). As a result, it can be assumed that most of the relative signal intensities of the *N*-and *O*-glycosylated peptide contains one core 1 *O*-glycan. The most abundant glycan composition of the tryptic glycopeptide is HexNAc_5_Hex_6_NeuAc_3_, which is most likely the *N*-glycan HexNAc_4_Hex_5_NeuAc_2_ considering the presence of a monosialylated core 1 *O*-glycan. In total, ∼68.9% of the glycans followed the composition HexNAc_5_Hex_6_ with different degrees of sialylation and with and without fucosylation. Likewise, 26.4% had the composition HexNAc_6_Hex_7_, 4.1% HexNAc_4_Hex_5_ and 0.6% HexNAc_7_Hex_8_. Including the likely presence of a core 1 *O*-glycan structure in each of these compositions, the glycosylation profile is like the one of Asn238, Asn253 and Asn352.

Glycosylation site Asn69 was characterized by Proteinase K-generated glycopeptides. Three different glycopeptides containing the *N*-glycosylation site Asn69 could be detected after Proteinase K-treatment, featuring non-fucosylated, di- and triantennary structures with two and three sialic acids. Because of the location of Asn69 close to six neighboring *O*-glycosylation sites (Ser63, Ser64, Thr67, Thr71, Thr72, Thr76) further mapping of the microheterogeneity in a relative manner was not possible.

The sixth *N*-glycosylation site Asn81 was identified as part of a heavily *O*-glycosylated peptide region after Pronase-treatment, followed by PNGase F *N*-glycan release and exoglycosidase treatment as described for *O*-glycopeptide identification. Most likely because of the large amount of *O*-glycans, the *N*-glycan release in this region was not complete, allowing the detection of the glycopeptide cluster with the peptide portion _79_TANTTDEPTTQPTTEPTTQPTIQPTQPTTQLPTDSPTQPTTGS_121_ featuring the glycan composition HexNAc_18–21_Hex_3_ (Fig. 4*A*, supplemental Fig. S12). The stepping-energy CID spectrum of this glycopeptide contained several diagnostic oxonium ions clearly indicating the presence of an *N*-glycan. However, because of the complexity of this glycopeptide and the application of exoglycosidases it was not possible to characterize the glycan microheterogeneity of Asn81.

Overall, the site-specific glycopeptide-based *N*-glycoform distribution was in good agreement with the total *N*-glycoform pattern determined by MALDI-TOF-MS of released *N*-glycans.

## DISCUSSION

In this study, we present for the first time a comprehensive site-specific glycoproteomic analysis of human C1-Inh by analyzing *N*- and *O*-glycopeptides next to released *N*-glycans with multimethodological mass spectrometric approaches.

Remarkably, we were able to detect up to 26 *O*-glycosylation events on C1-Inh. Of these, nine were assigned to a specific site in the N-terminal domain (Ser31, Thr47, Thr48, Ser63, Ser64, Thr67, Thr71, Thr72, Thr76). Another *O*-glycan could be assigned to either Thr27 or Ser28, leaving some ambiguity. With respect to the other detected *O*-glycans, we were able to identify a heavily *O*-glycosylated region (Thr_82_-Ser_121_) with up to 16 occupied *O*-glycosylation sites out of 18 possible sites by tandem MS. This shows overall very high *O*-glycosylation site occupancy of this mucin-type region.

Our findings are perfectly in line with previous studies on C1-inhibitor *O*-glycosylation, but go way beyond those reports: *O*-glycosylation sites Thr47 and Thr48 have been reported earlier in human plasma, cerebrospinal glycoproteins and human urine containing core-1 *O*-glycans (10, 13, 25) and were confirmed in our study. Likewise, Ser31 was confirmed from human plasma (13). Furthermore, Ser64 and Ser71 are known from amino acid sequencing studies (11) and were for the first time confirmed by our mass spectrometric study. The occupation of the sites Thr83, Thr88, Thr92, and Thr96 has previously been revealed by amino acid sequencing, and in addition Bock *et al.* also suggested the *O*-glycosylation of Thr99, Thr106, Thr107, Thr111, Thr115, Thr118, and Thr119, however, they could not provide unequivocal evidence (11). Additionally, the peptide portions _113_SPTQPTTGSF_121_ and _113_SPTQPTTGSFCPGPVTL_128_ have been recently identified with two and one O-glycan attached, respectively. These findings are largely in-line with our mass spectrometric data that demonstrated occupation of up to 16 of the 18 possible *O*-glycosylation sites in the region Thr_82_-Ser_121_. Hence, the MS(/MS) analysis of Thr_82_-Ser_121_ showed for the first time the excessive *O*-glycosylation of this region. Notably, to the best of our knowledge the *O*-glycosylation sites Ser63, Thr67, Thr72, Thr76 as well as the *O*-glycosylation region Thr27/Ser28 are reported for the first time. In a recent study on plasma *O*-glycosylation also Ser36 was identified as an *O*-glycosylation site (13), which was not confirmed by our data using the purified glycoprotein. However, we cannot exclude the glycosylation of this site.

In the current study, we mainly observe mono-, di-, and non-sialylated core 1 *O*-glycans and smaller amounts of core 2 *O*-glycans for the glycosylation sites Thr47/Thr48. It might be also possible that the other *O*-glycosylation sites carry smaller amounts of core 2 *O*-glycans, which were not detected.

These findings can be only compared with Ser31, Thr47 and Thr48, because these sites have been identified before by MS (10, 13, 25). However, in these studies the glycopeptides were captured using hydrazine chemistry, which included acid hydrolysis to release *O*-glycopeptides, or after sialidase treatment of the glycopeptides. Using the hydrazine chemistry approach confirmed that the *O*-glycopeptides contained sialic acid but further information about the glycan microheterogeneity was lost. In all three studies only core 1-containing glycopeptides were identified.

The *N*-glycosylation sites of C1-Inh have been mapped by mass spectrometry (25–31), as well as other analytical approaches in various studies (4, 8, 12, 32). In agreement with literature, we confirmed the glycosylation of all six *N*-glycosylation sites. Perkins *et al.* and Strecker *et al.*, reported that C1-Inh carried disialylated complex-type *N*-glycans (8, 12) and small amounts of tri- and tetraantennary glycan structures (8). This is in accordance with the major structure detected in our study. Similarly, small amounts of tri- and tetra-antennary glycan structures have been detected by Perkins *et al.* Hitherto, knowledge on site-specific *N*-glycosylation of C1-Inh has been very limited. A previous report detected solely diantennary structures on Asn352 (31), whereas we also detected small amounts of less than 3% of triantennary glycan structures at this site.

The role of C1-Inh glycosylation has been investigated and discussed with regards to its functional activity in a few studies. The high degree of sialylation can prolong serum half-life as desialylation of the protein resulted in faster clearance from blood in rabbits, supposedly because of the asialoglycoprotein receptor in the liver (33). However, deglycosylation of human C1-Inh with PNGase F, *O*-glycanase, or both had no detectable impact on its C1s protease inhibition activity (34, 35). Different observations were made for the involvement of C1-Inh glycosylation in the inhibition of kallikrein. Although the desialylated and de-*N*-glycosylated human C1-Inh retains its ability to complex kallikrein, the further *O*-glycanase-treated protein species lost this feature (35). These results suggest the contribution of N-terminal *O*-glycosylation in C1-Inh protease inhibition in the contact system. In contrary, studies investigating the protease inhibition activity of recombinant WT C1-Inh and constructs with deletion of the first 98 or 120 amino acid, representing the highly glycosylated N-terminal domain, showed the same interaction kinetics with C1s, kallikrein or factor XIIa (amino acid numbering including the signal peptide; recombinant expression in *P. pastoris* and *E. coli*) (36, 37).

The N terminus has been described to be a “rod-like” domain, likely because of the presence of the glycans (8, 11, 38). In fact, the N-terminal domain, either because of its size and/or charge, has been suggested to interfere with the interaction of C1-Inh with cell surface bound kallikrein and factor XIIa (39, 40). It may be hypothesized that the extensive *N*- and *O*-glycosylation increases the overall and local size, charge, and hydrophilicity as well as thermodynamic stability of the N-terminal domain, thereby modifying the physicochemical properties of C1-Inh. Moreover, we found that the highly *O*-glycosylated region with up to 12–16 occupied sites was resistant to protease treatment, demonstrating that glycosylation can prevent C1-Inh from proteolytic degradation.

In conclusion, we here applied a powerful approach for in-depth site-specific characterization of C1-Inh glycosylation revealing 10 *O*-glycosylation sites carrying mainly core-1 type *O*-glycans, with five of them being novel. In addition, we identified a heavily *O*-glycosylated portion of C1-Inh spanning from Thr_82_-Ser_121_ with up to 16 *O*-glycans attached. Likewise, we covered all six *N*-glycosylation sites of C1-Inh by site-specific glycosylation analysis.

This extensive and specific information on C1-Inh glycosylation will help to better understand existing functional studies and it is essential for future targeted studies to investigate the role of glycosylation of this plasma glycoprotein. Consequently, we elucidated plasma-derived human C1-Inh glycosylation, which will be important to further evaluate the glycosylation profiles of recombinantly expressed C1-Inh.

## DATA AVAILABILITY

All glycopeptide tandem MS spectra are available under the following link: ftp://massive.ucsd.edu/MSV000081096.

## Supplementary Material

Supplemental Data

## Footnotes

^1^ The abbreviations used are:

AmBiC: ammonium bicarbonate
C1-Inh: C1-Inhibitor
ENeuAc: α2,6-linked N-acetylneuraminic acid
EDC: 1-ethyl-3-(3-dimethylaminopropyl)carbodiimide hydrochloride
ETD: electron-transfer dissociation
FA: formic acid
Fuc: fucose
GalNAc: N-acetylgalactosamine
GlcNAc: N-acetylglucosamine
Hereditary angioedema: HAE
Hex: hexose
HexNAc: N-acetylhexosamine
HILIC: hydrophilic interaction liquid chromatography
HOBt: hydroxybenzotriazol hydrate
IAA: iodoacetamide
IPQ score: isotopic pattern quality score
IT: ion trap
LNeuAc: α2,3-linked N-acetylneuraminic acid
Na_2_HPO_4_x2H_2_O: sodium hydrogen phosphate dihydrate
NaOH: sodium hydroxide
NeuAc: N-acetylneuraminic acid
NP-40: Nonidet P-40
PGC: porous graphitized carbon
PNGase F: peptide-N-glycosidase F
RP: reversed-phase
sDHB: 2-hydroxy-5-methoxy-benzoic acid and 2,5-dihydroxybenzoic acid
Serpin: serine protease inhibitor
SPE: solid phase extraction.
