# Supplementary material for: N- and O-glycosylation Analysis of Human C1-inhibitor Reveals Extensive Mucin-type O-Glycosylation
Source: Mol Cell Proteomics. 2017 Dec 12;17(6):1225–38. doi: 10.1074/mcp.RA117.000240 (PMC5986245; doi:10.1074/mcp.RA117.000240)
Supplement: Supplemental Data [file supp_17_6_1225__index.html]

N- and O-glycosylation analysis of human C1-inhibitor reveals extensive mucin-type O-glycosylation — Human C1-Inhibitor glycosylation — N- and O-glycosylation Analysis of Human C1-inhibitor Reveals Extensive Mucin-type O-Glycosylation — Human C1-Inhibitor Glycosylation — Supplemental Data 

# N- and *O*-glycosylation Analysis of Human C1-inhibitor Reveals Extensive Mucin-type *O*-Glycosylation

## Supplemental Data

- Supplementary information - Supplemental methods information, glycopeptide and glycan spectra
- Supplemental tables - Supplemental tables with all identified glycopeptides, glycans and their corresponding relative intensities.
