# Supplementary material for: N- and O-glycosylation Analysis of Human C1-inhibitor Reveals Extensive Mucin-type O-Glycosylation
Source: Mol Cell Proteomics. 2017 Dec 12;17(6):1225–38. doi: 10.1074/mcp.RA117.000240 (PMC5986245; doi:10.1074/mcp.RA117.000240)
Supplement: Supplemental Data [file supp_RA117.000240_4850_1_supp_27638_kzk3vp.pdf]

Supplemental Information associated with manuscript:

N- and *O*-glycosylation analysis of human C1-inhibitor reveals extensive mucin-type *O*-glycosylation

Kathrin Stavenhagen[1,2+*], *H. Mehmet Kayili*[2,3,4,5+], *Stephanie Holst*[1], *Carolien A. M. Koeleman*[1], *Ruchira Engel*[6,7], *Diana Wouters*[6,7], *Sacha Zeerleder*[6,7], *Bekir Salih*[5], *Manfred Wuhrer*[1,2]

[1] Center for Proteomics and Metabolomics, Leiden University Medical Center, Leiden, The Netherlands

[2] Division of BioAnalytical Chemistry, VU University Amsterdam, Amsterdam, The Netherlands

[3] Department of Chemistry, Çankırı Karatekin University, Çankırı, Turkey

[4] Department of Nutrition and Dietetics, Karabuk University, Karabuk, Turkey

[5] Department of Chemistry, Hacettepe University, Ankara, Turkey

[6] Department of Immunopathology, Sanquin Research and Landsteiner Laboratory of the AMC, Amsterdam, The Netherlands

[7] Department of Hematology, Academic Medical Center, University of Amsterdam, The Netherlands

[+] Authors equally contributed to this work

* Corresponding author

Materials and Methods

C18-LC-ESI-IT-MS/MS

Pronase-treated (glyco)peptides of C1-Inh obtained from in-gel digestions were diluted 10 times with water and 4 µL were injected into a C18-LC-ESI-IT-MS/MS system consisting of an Ultimate 3000 RSLCnano system (Thermo Scientific, Sunnyvale, CA) coupled to an amazonSpeed ion trap (Bruker Daltonics). Data analysis was performed using DataAnalysis 4.2 (Bruker Daltonics).

Samples were loaded on a precolumn (Acclaim PepMap100 C18 column, 100 µm × 2 cm, 5 µm, 100 Å, Thermo Scientific) prior to separation on an Acclaim PepMap RSLC nano-column (75 µm × 15 cm, 2 µm, 100 Å, Thermo Scientific). A flow rate of 300 nL/min was applied in a multistep linear gradient (t = 0-5 min, c(B) = 3%; t = 35 min, c(B) = 27%; t = 40-45 min, c(B) = 70%; t = 46-58 min, c(B) = 3% with solvent A (0.1% FA in water) and solvent B (95% ACN and 5% water).

All analyses were performed in positive ion mode using a CaptiveSpray nanoBooster (Bruker Daltonics) with acetonitrile-enriched nitrogen at 0.2 bar. The source parameters were set to a dry gas flow of 3 L/min at 150°C and a capillary voltage of 1300 V. MS acquisition was performed in enhanced resolution mode within a *m/z*-range from *m/z* 400 to *m/z* 2000 or *m/z* 600 to *m/z* 1800. The maximum accumulation time was 200 ms, the ICC target was set to 200000 and SPS to *m/z* 1100 at a trap drive level of 100%. ETD MS/MS spectra were acquired from *m/z* 140 to *m/z* 2200 for a selected list of precursors. The ICC target was set to 100000.

Released *N*-glycan analysis

In-gel PNGase F treatment for the analysis of released *N*-glycans

In-gel digestions were performed as previously described [1] with minor modifications. Briefly, bands of interest were cut into pieces, and transferred to Eppendorf tubes. Gel pieces were sequentially washed with washing buffer (25 mM sodium bicarbonate, pH 8) and dehydrated with ACN. Proteins were reduced in-gel by incubating for 30 min at 56°C in reduction buffer (10 mM DTT in washing buffer) and dehydrated with ACN prior to in-gel cysteine alkylation for 20 min at RT in alkylation buffer (55 mM IAA in washing buffer) protected from light. Samples were repeatedly washed and dehydrated with washing buffer and ACN, respectively, until gel pieces were completely de-stained. Gel particles were dried in a centrifugal vacuum concentrator at 30°C for a maximum of 5 min. Subsequently, 20 µL to 30 µL PNGase F solution (2 U in 2% NP-40 and 2.5xPBS) were added. Samples were kept on ice for 1 h to let the gel adsorb the buffer and enzymatic cleavage was allowed to take place overnight at 37°C. Next,

the supernatant containing *N*-glycans was collected, 20 µL of water were added to the gel pieces and after 20 min of sonication the supernatant was added to the first extraction. The combined supernatants containing the *N*-glycans were further processed as described below.

Derivatization of released *N*-glycans and hydrophilic interaction liquid chromatography (HILIC)-solid phase extraction (SPE) glycan enrichment

Released *N*-glycans from the in-gel PNGase F treatment were derivatized by ethyl esterification as described in Reiding *et al*. [2] with small adaptations. Shortly, 2 µL of released *N*-glycans were added to 20 µL of ethylation reagent (0.25 M EDC and 0.25 M HOBt in ethanol, 1:1) in quadruplicate, and incubated for 1 h at 37°C (in desiccator protected from evaporation). Subsequently, 20 µL ACN were added and the mixture was incubated at -20°C for 30 min. Samples were let to reach room temperature prior to glycan purification by HILIC-SPE modified from a protocol described previously [3]. Pipette tips of 20 µL volume were packed with 3 mm cotton thread, washed by pipetting 3 x 20 µL water, followed by equilibration with 3 x 20 µL 85% ACN. Samples were loaded by carefully pipetting up-and-down for 30 times. Unbound material was removed by sequential pipetting of 3 x 20 µL 85% ACN/1% TFA and 3 x 20 µL 85% ACN, followed by elution of *N*-glycans in 10 µL water.

MALDI-TOF/TOF-MS/MS analysis

For mass spectrometric analysis, 5 µL of released, derivatized, and purified *N*-glycans were spotted onto an anchor chip MALDI target plate (Bruker Daltonics, Bremen, Germany) and co-crystallized with 1 µL of 5 mg/mL sDHB in 50% ACN/50% water containing 1 mM NaOH. Samples were allowed to dry at room temperature. MALDI-TOF-MS spectra were acquired using an UltrafleXtreme mass spectrometer (Bruker Daltonics) in positive ion reflector mode, controlled by flexControl 3.4 software Build 119 (Bruker Daltonics). The instrument was externally calibrated using a peptide calibration kit (Bruker Daltonics). Spectra were obtained over a mass window of *m/z* 1000 to *m/z* 5000 with suppression up to *m/z* 900 for a total of 20000 shots (2000 Hz laser frequency, 200 shots per raster spot during complete random walk). Tandem mass spectrometry (MALDI-TOF/TOF-MS/MS) was performed for structural elucidation via fragmentation in gas-off TOF/TOF mode.

Data processing and evaluation of MALDI-TOF-MS spectra

The *N*-glycan profiles obtained by MALDI-TOF-MS were internally re-calibrated on the following peaks *m/z* 1982.700 (hexose $(Hex)_5$ *N*-acetylhexosamine $(HexNAc)_4$(α2,6)$NeuAc_1$), *m/z* 2255.793 ($Hex_5HexNAc_4$(α2,3)$NeuAc_1$(α2,6)$NeuAc_1$), *m/z* 2301.835 ($Hex_5HexNAc_4$(α2,6)$NeuAc_2$), *m/z* 2447.893 ($Hex_5HexNAc_4$(α2,6)$NeuAc_2$ fucose$(Fuc)_1$), *m/z* 2940.052 ($Hex_6HexNAc_5$(α2,3)$NeuAc_1$(α2,6)$NeuAc_2$), *m/z* 3086.113 ($Hex_6HexNAc_5$(α2,3)$NeuAc_1$(α2,6)$NeuAc_2Fuc_1$), *m/z* 3532.227 ($Hex_7HexNAc_6$(α2,3)$NeuAc_3$(α2,6)$NeuAc_1$), and *m/z* 3578.269 ($Hex_7HexNAc_6$(α2,3)$NeuAc_2$(α2,6)$NeuAc_2$) using the cubic enhanced algorithm in FlexAnalysis Software (Version 3.3 build 65; Bruker Daltonics). Spectra were further smoothed (Savitzky Golay algorithm, peak width: *m/z* 0.06, 4 cycles) and baseline-corrected (Tophat algorithm). A composition list for targeted data extraction was generated comprising peaks with signal/noise>3, proper isotopic pattern as judged by visual inspection, and matching glycan composition determined with GlycoWorkbench 2.1 stable build 140 (European Carbohydrates DataBase project; http://www.eurocarbdb.org/) with accuracy of 0.3 Da.

The calibrated spectra, together with the reviewed composition list were applied to an in-house developed software for automated data processing MassyTools 1.0 [4], which extracted the area under the curve for each peak of the mass list. Background defined as the lowest area observed in a *m/z*-window of 20 Th around the analyte was subtracted from intensities of all isotopic peaks. Furthermore, peaks in the spectra with negative intensity values were set to an intensity value of zero and areas under the curve were normalized to a total relative intensity of 100% for each spectrum. At the end, only glycan compositions which have been confirmed by MS/MS and their directly related compositions (± one monosaccharide) were taken into account for relative quantitation.

Next, derived glycosylation traits such as α2,6-sialylation, α2,3-sialylation, fucosylation, antenna fucosylation (defined as amount of fucoses equal or higher than two) were calculated by summing the relative intensities of the relevant peaks, and averaged within the four technical replicates. GraphPad Prism Version 5.04 (2010, GraphPad Software, Inc., La Jolla, CA) was used for visualization.

Methods and materials

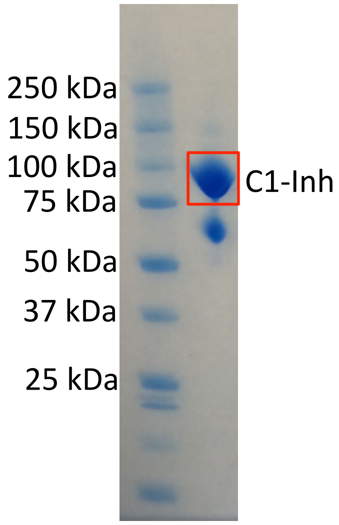

Figure S1: SDS-PAGE of C1-Inh. The highlighted band was used for glycopeptide and glycan analysis.

Results

Site-specific determination of the C1-Inh *O*-glycosylation sites

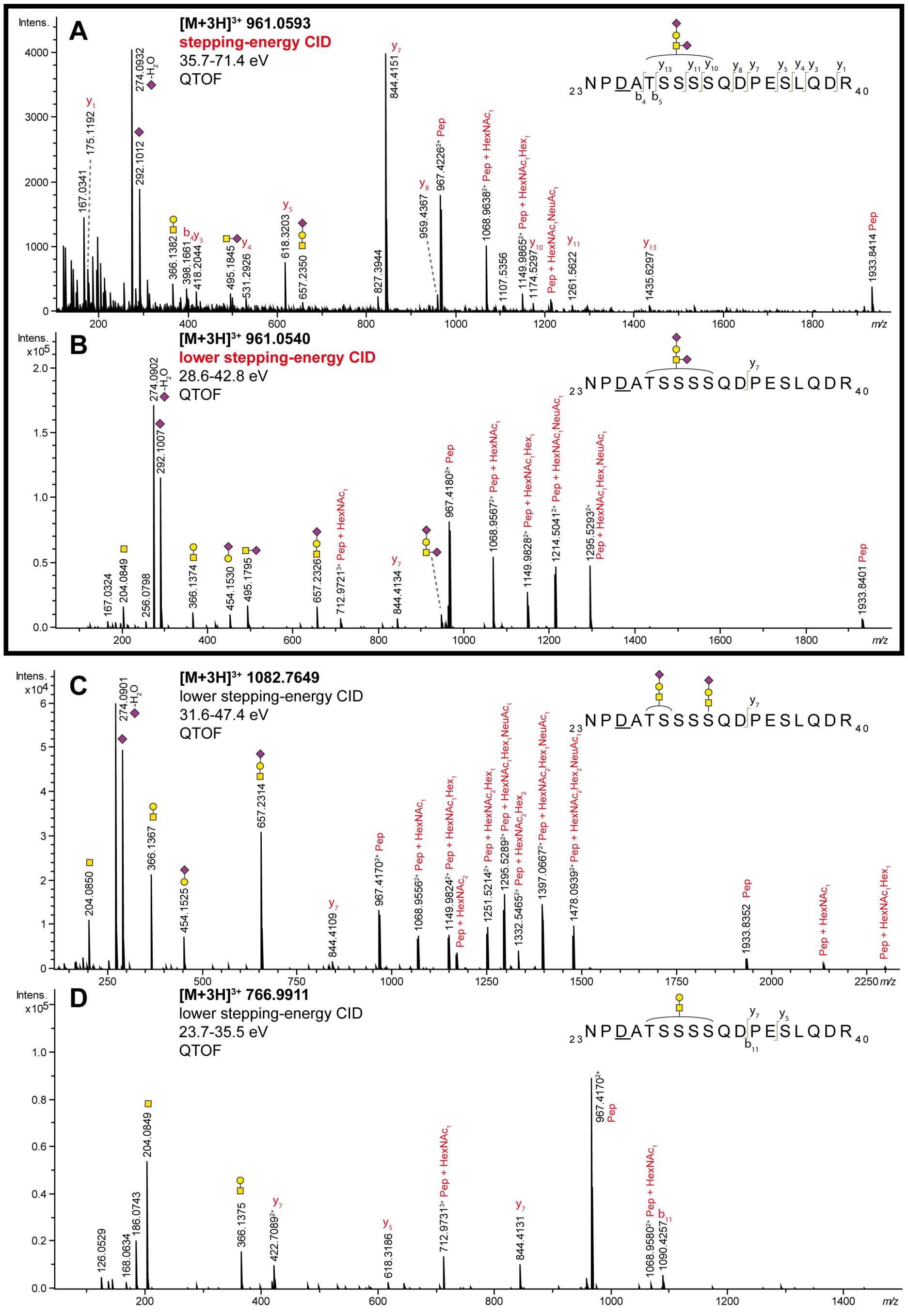

Figure S2: Stepping-energy CID spectra of C1-Inhibitor glycopeptides with the peptide portion $_{23}$NPDATSSSSQDPESLQDR$_{40}$ (D indicating deamidation after PNGase F treatment) covering the glycosylation sites Thr27/Ser28 and Ser31. (**A**) Stepping-energy CID spectrum of the glycopeptide with the glycan portion $HexNAc_1Hex_1NeuAc_2$. (**B**) Lower stepping-energy CID spectrum (stepping energy is set to 60% and 40% each 20% and 80% of the time, respectively) of the glycopeptide with the glycan portion $HexNAc_1Hex_1NeuAc_2$. (**C**) Lower stepping-energy CID spectrum of the glycopeptide with the glycan portion $HexNAc_1Hex_1NeuAc_1$ and $HexNAc_1Hex_1NeuAc_1$. (**D**) Lower stepping-energy CID spectrum of the glycopeptide with the glycan portion $HexNAc_1Hex_1$.

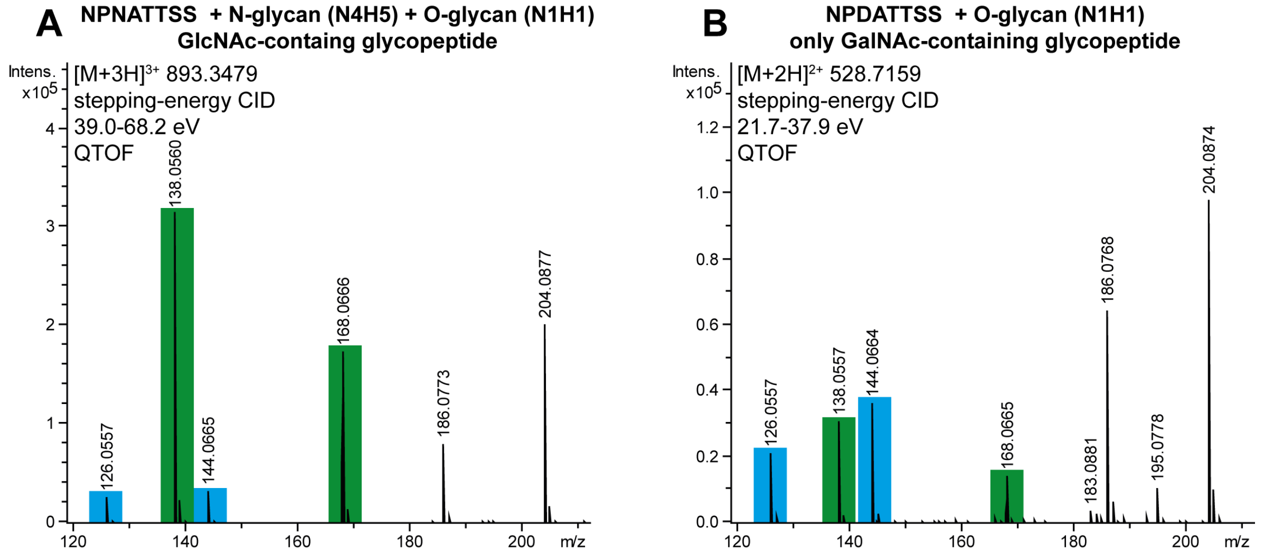

Figure S3: Zoom-in of the lower mass region HexNAc-related oxonium ions in stepping-energy CID of the glycopeptides with the peptide portion NPNATTSS and with the glycan portion (**A**) $HexNAc_4Hex_5$ + $HexNAc_1Hex_1$ and (**B**) $HexNAc_1Hex_1$. A higher ratio of *m/z* 138 ($[HexNAc\text{-}CH_6O_3]^+$) + *m/z* 168 ($[HexNAc\text{-}2H_2O]^+$) compared to *m/z* 126 ($[HexNAc\text{-}C_2H_6O_3]^+$) + *m/z* 144 ($[HexNAc\text{-}C_2H_4O_2]^+$) are diagnostic for a GlcNAc-containing glycopeptide. More equal amounts of *m/z* 138 + *m/z* 168 compared to *m/z* 126 + *m/z* 144 are indicative for only GalNAc-containing glycopeptides[5]. Since the glycopeptide in (**A**) contains a *N*-glycan moiety with GlcNAc residues the signal intensities for *m/z* 138 ($[HexNAc\text{-}CH_6O_3]^+$) + *m/z* 168 ($[HexNAc\text{-}2H_2O]^+$) are higher compared to *m/z* 126 ($[HexNAc\text{-}C_2H_6O_3]^+$) + *m/z* 144 ($[HexNAc\text{-}C_2H_4O_2]^+$). The same glycopeptide which only contains a core 1 *O*-glycan features an almost equal ratio of *m/z* 138 ($[HexNAc\text{-}CH_6O_3]^+$) + *m/z* 168 ($[HexNAc\text{-}2H_2O]^+$) are higher compared to *m/z* 126 ($[HexNAc\text{-}C_2H_6O_3]^+$) + *m/z* 144 ($[HexNAc\text{-}C_2H_4O_2]^+$).

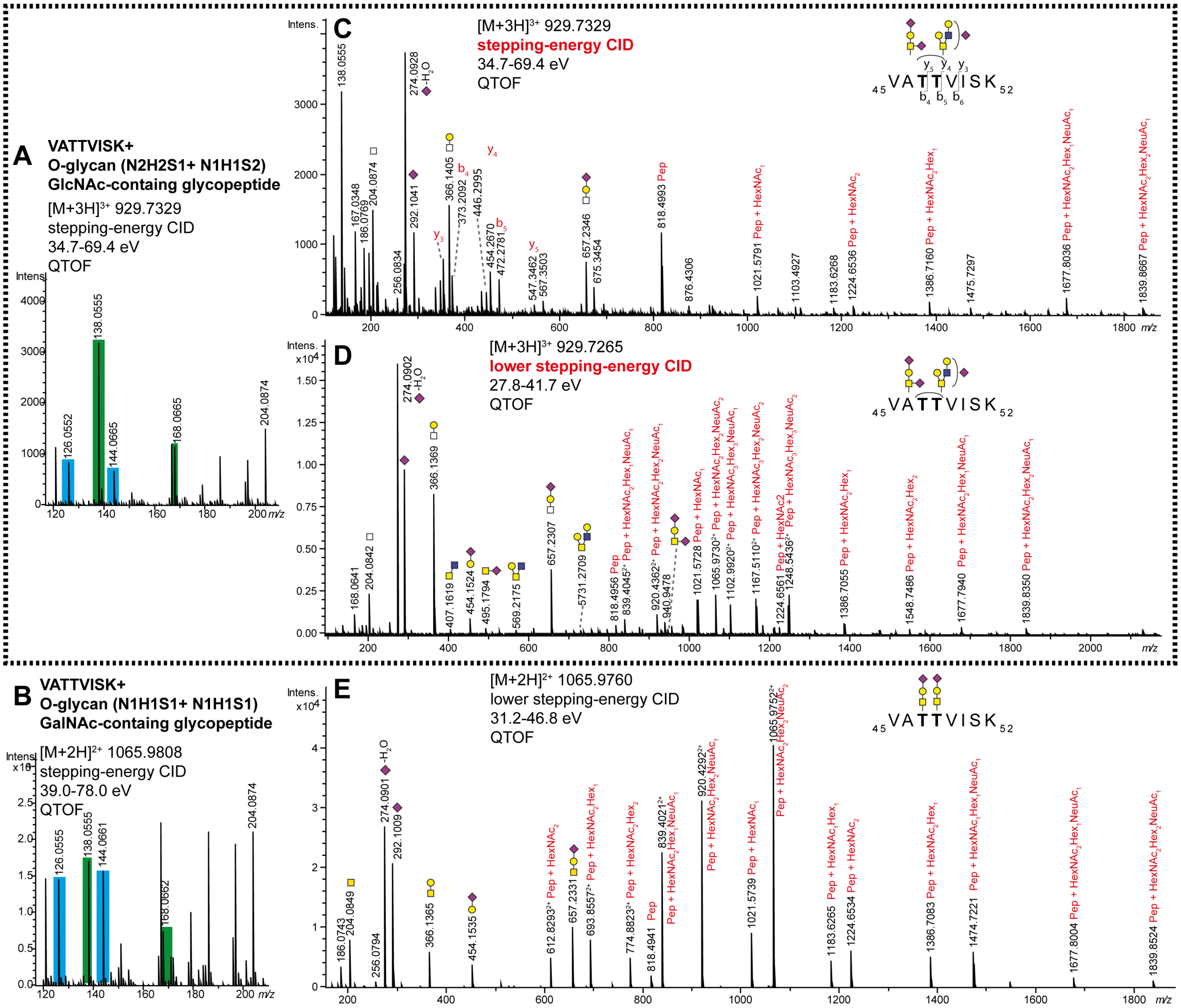

Figure S4: Stepping-energy CID spectra of C1-Inhibitor glycopeptides with the peptide portion $_{45}$VATTVISK$_{52}$ covering the glycosylation sites Thr47 and Thr48. (**A**) Zoom-in of the lower mass region HexNAc-related oxonium ions in stepping-energy CID of the glycopeptide with the glycan portion $HexNAc_1Hex_1NeuAc_2$ and $HexNAc_2Hex_2NeuAc_1$. A higher ratio of *m/z* 138 ($[HexNAc\text{-}CH_6O_3]^+$) + *m/z* 168 ($[HexNAc\text{-}2H_2O]^+$) compared to *m/z* 126 ($[HexNAc\text{-}C_2H_6O_3]^+$) + *m/z* 144 ($[HexNAc\text{-}C_2H_4O_2]^+$) are diagnostic for a GlcNAc-containing glycopeptide. More equal amounts of *m/z* 138 + *m/z* 168 compared to *m/z* 126 + *m/z* 144 are indicative for only GalNAc-containing glycopeptides. This different ratio indicates that the glycopeptide contains a GlcNAc. (**B**) Zoom-in of the lower mass region HexNAc-related oxonium ions in stepping-energy CID of the glycopeptide with the glycan portion $HexNAc_1Hex_1NeuAc_1$ and $HexNAc_1Hex_1NeuAc_1$. The almost equal ratio indicates that the glycopeptide contains only GalNAc. (**C**) Stepping-energy CID spectrum of the glycopeptide with the glycan portion $HexNAc_1Hex_1NeuAc_2$ and $HexNAc_2Hex_2NeuAc_1$. (**D**) Lower stepping-energy CID spectrum (stepping energy is set to 60% and 40% each 20% and 80% of the time, respectively) of the glycopeptide with the glycan portion $HexNAc_1Hex_1NeuAc_2$ and $HexNAc_2Hex_2NeuAc_1$. (**E**) Lower stepping-energy CID spectrum of the glycopeptide with the glycan portion $HexNAc_1Hex_1NeuAc_1$ and $HexNAc_1Hex_1NeuAc_1$.

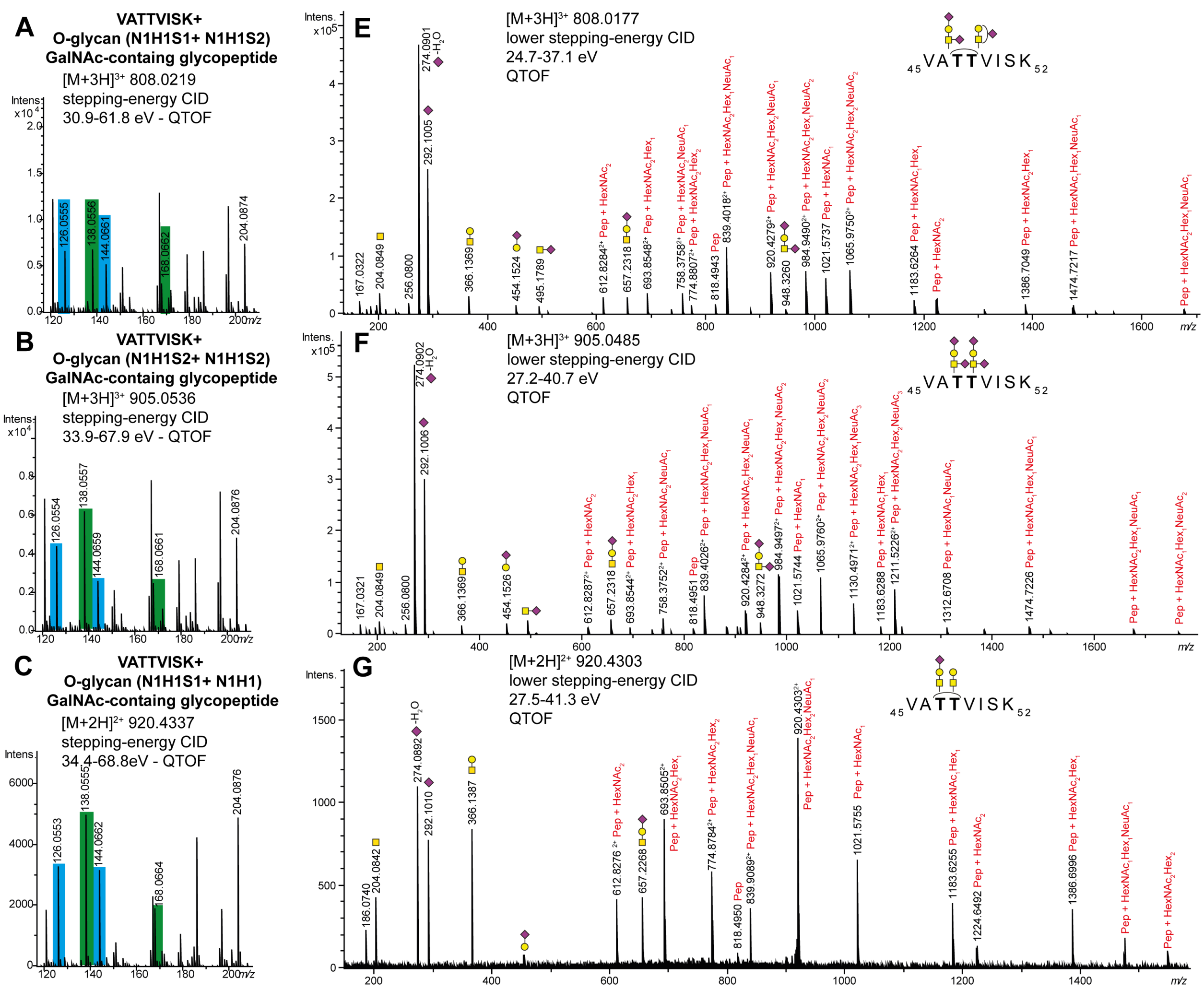

Figure S5: Stepping-energy CID spectra of C1-Inhibitor glycopeptides with the peptide portion $_{45}$VATTVISK$_{52}$ covering the glycosylation sites Thr47 and Thr48. (**A**) Zoom-in of the lower mass region HexNAc-related oxonium ions in stepping-energy CID of the glycopeptide with the glycan portion $HexNAc_1Hex_1NeuAc_2$ and $HexNAc_1Hex_1NeuAc_1$. A higher ratio of *m/z* 138 ($[HexNAc\text{-}CH_6O_3]^+$) + *m/z* 168 ($[HexNAc\text{-}2H_2O]^+$) compared to *m/z* 126 ($[HexNAc\text{-}C_2H_6O_3]^+$) + *m/z* 144 ($[HexNAc\text{-}C_2H_4O_2]^+$) are diagnostic for a GlcNAc-containing glycopeptide, more equal amounts of *m/z* 138 + *m/z* 168 compared to *m/z* 126 + *m/z* 144 are indicative for only GalNAc-containing glycopeptides. The almost equal ratio indicates that the glycopeptide contains a GlcNAc. (**B**) Zoom-in of the lower mass region HexNAc-related oxonium ions in stepping-energy CID of the glycopeptide with the glycan portion $HexNAc_1Hex_1NeuAc_2$ and $HexNAc_1Hex_1NeuAc_2$. The almost equal ratio indicates that the glycopeptide contains only GalNAc. (**C**) Zoom-in of the lower mass region HexNAc-related oxonium ions in stepping-energy CID of the glycopeptide with the glycan portion $HexNAc_1Hex_1NeuAc_1$ and $HexNAc_1Hex_1$. The almost equal ratio indicates that the glycopeptide contains only GalNAc. (**D**) Lower stepping-energy CID spectrum (stepping energy is set to 60% and 40% each 20% and 80% of the time, respectively) of the glycopeptide with the glycan portion $HexNAc_1Hex_1NeuAc_2$ and $HexNAc_1Hex_1NeuAc_1$. (**E**) Lower stepping-energy CID spectrum of the glycopeptide with the glycan portion $HexNAc_1Hex_1NeuAc_2$ and $HexNAc_1Hex_1NeuAc_2$. (**F**) Lower stepping-energy CID spectrum of the glycopeptide with the glycan portion $HexNAc_1Hex_1NeuAc_1$ and $HexNAc_1Hex_1$.

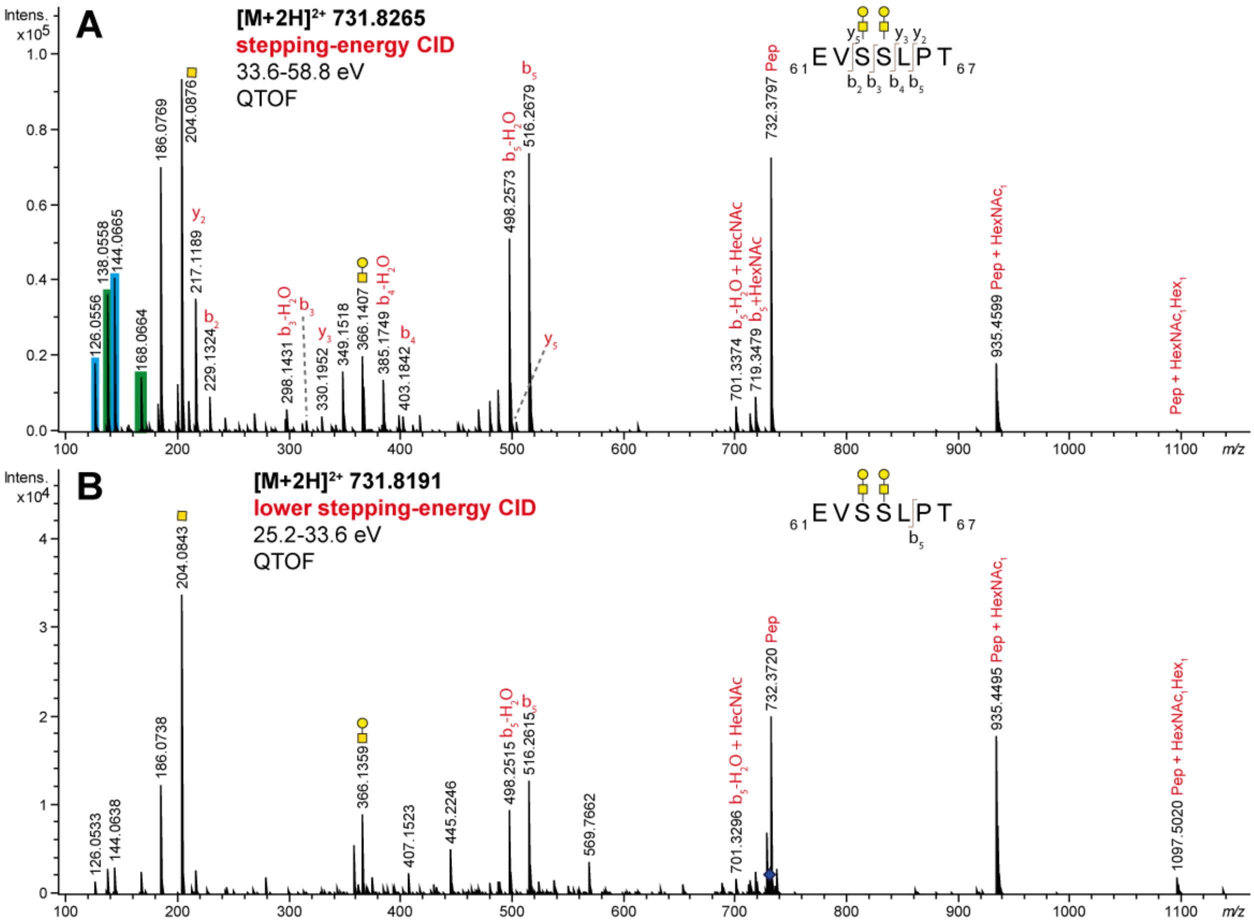

Figure S6: Stepping-energy CID spectra of C1-Inhibitor glycopeptides with the peptide portion $_{61}$EVSSLPT$_{67}$ covering the glycosylation sites Ser63 and Ser64. (**A**) Stepping-energy CID spectrum of the glycopeptide with the glycan portion HexNAc$_1$Hex$_1$ and HexNAc$_1$Hex$_1$. The lower mass region HexNAc-related oxonium ions in stepping-energy CID of the glycopeptide feature and almost equal ratio of *m/z* 138 ([HexNAc-CH_6O_3]$^+$) + *m/z* 168 ([HexNAc-$2H_2O$]$^+$) compared to *m/z* 126 ([HexNAc-$C_2H_6O_3$]$^+$) + *m/z* 144 ([HexNAc-$C_2H_4O_2$]$^+$) indicating that the glycopeptide contains only GalNAc. This further supports the conclusion that this glycopeptide contains two individual core 1 O-glycans attached to Ser63 and Ser64, as identified by ETD. (**B**) Lower stepping-energy CID spectrum (stepping energy is set to 60% and 40% each 20% and 80% of the time, respectively) of the glycopeptide with the glycan portion HexNAc$_1$Hex$_1$ and HexNAc$_1$Hex$_1$. Note: The peak with the *m/z* 407.1523 does not correspond the exact mass of 2 HexNAcs, which would lead to a different assignment of the glycan portion.

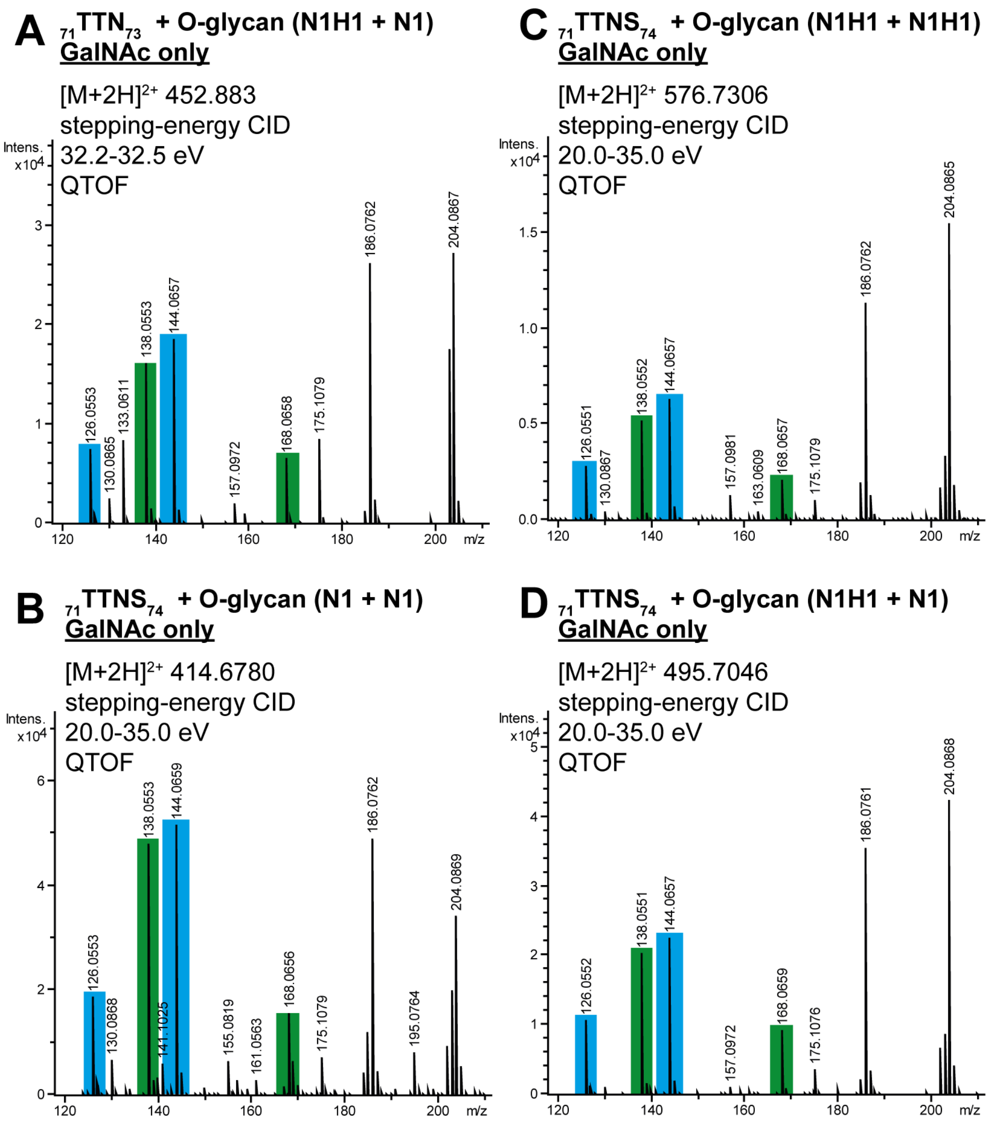

Figure S7: Zoom-in of the lower mass region HexNAc-related oxonium ions in stepping-energy CID of the O-glycosylation site Thr71 and Thr72. A higher ratio of *m/z* 138 ($[HexNAc\text{-}CH_6O_3]^+$) + *m/z* 168 ($[HexNAc\text{-}2H_2O]^+$) compared to *m/z* 126 ($[HexNAc\text{-}C_2H_6O_3]^+$) + *m/z* 144 ($[HexNAc\text{-}C_2H_4O_2]^+$) are diagnostic for a GlcNAc-containing glycopeptide. More equal amounts of *m/z* 138 + *m/z* 168 compared to *m/z* 126 + *m/z* 144 are indicative for only GalNAc-containing glycopeptides[5]. All glycopeptides feature a nearly equal ratio *m/z* 138 + *m/z* 168 compared to *m/z* 126 + *m/z* 144, indicating mainly GalNAc-containg core 1 O-glycans. (**A**) The glycopeptide $_{71}$TTN$_{73}$ with HexNAc$_2$Hex$_1$ (**B**) The glycopeptide $_{71}$TTNS$_{72}$ with HexNAc$_2$. (**C**) The glycopeptide $_{71}$TTNS$_{74}$ with HexNAc$_2$Hex$_1$. (**D**) The glycopeptide $_{71}$TTNS$_{72}$ with HexNAc$_2$Hex$_1$.

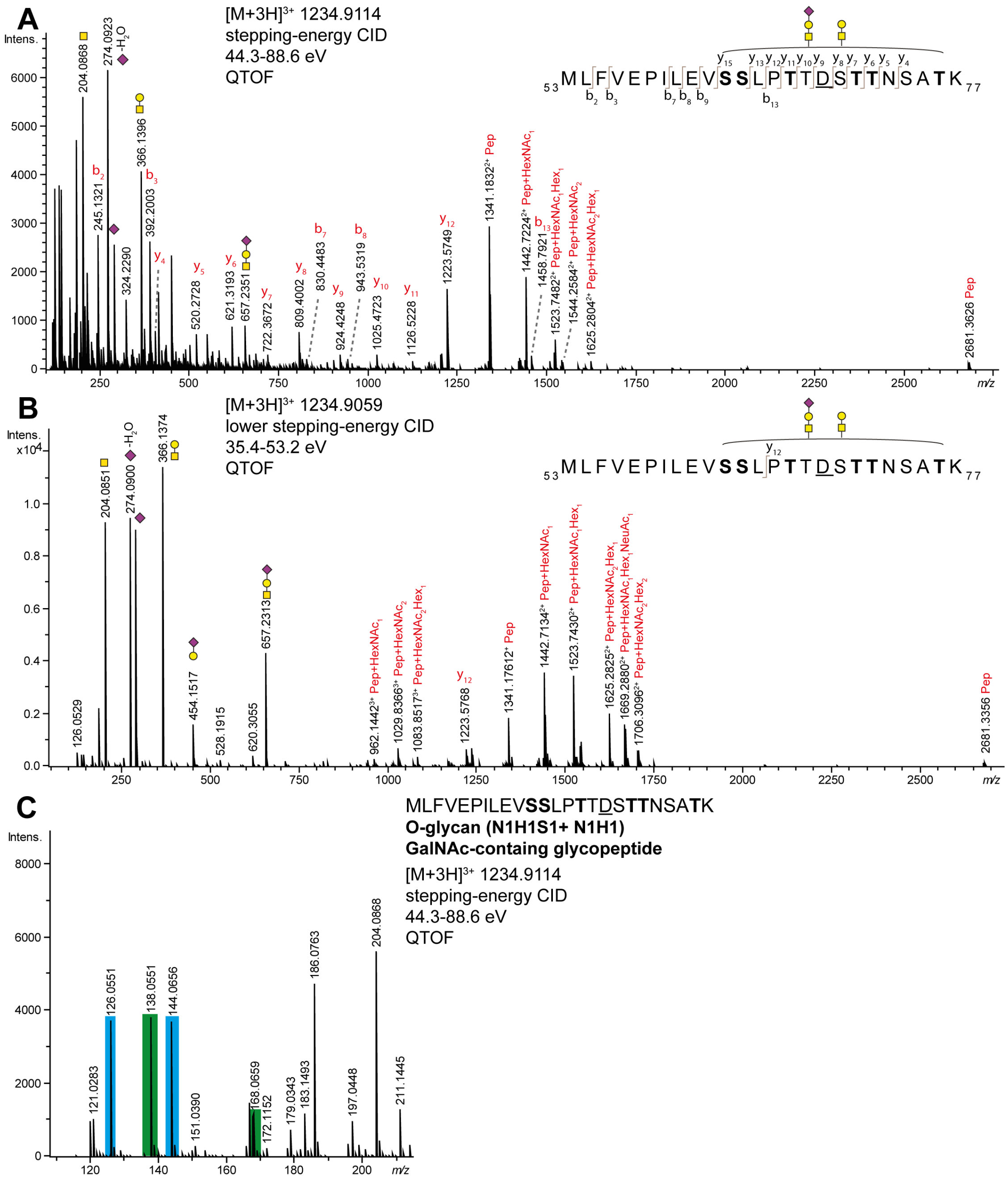

Figure S8: Stepping-energy CID spectra of C1-Inhibitor glycopeptides with the peptide portion $_{53}$MLFVEPILEVSSLPTTDSTT NSATK$_{77}$ covering the glycosylation sites Ser63, Ser64, Thr67, Thr71, Thr72, Thr76. (**A**) Stepping-energy CID spectrum of the glycopeptide with the glycan portion HexNAc$_1$Hex$_1$NeuAc$_1$ and HexNAc$_1$Hex$_1$. (**B**) Lower stepping-energy CID spectrum (stepping energy is set to 60% and 40% each 20% and 80% of the time, respectively) of the glycopeptide with the glycan portion HexNAc$_1$Hex$_1$NeuAc$_1$ and HexNAc$_1$Hex$_1$. (**C**) Zoom-in of the lower mass region HexNAc-related oxonium ions in stepping-energy CID of the glycopeptide with the glycan portion HexNAc$_1$Hex$_1$NeuAc$_2$ and HexNAc$_1$Hex$_1$NeuAc$_1$. A higher ratio of *m/z* 138 ([HexNAc-CH_6O_3]$^+$) + *m/z* 168 ([HexNAc-$2H_2O$]$^+$) compared to *m/z* 126 ([HexNAc-$C_2H_6O_3$]$^+$) + *m/z* 144 ([HexNAc-$C_2H_4O_2$]$^+$) are diagnostic for a GlcNAc-containing glycopeptide, more equal amounts of *m/z* 138 + *m/z* 168 compared to *m/z* 126 + *m/z* 144 are indicative for only GalNAc-containing glycopeptides. The almost equal ratio indicates that the glycopeptide contains a GlcNAc. Lower stepping-energy CID spectrum of the glycopeptide with the glycan portion HexNAc$_1$Hex$_1$NeuAc$_2$ and HexNAc$_1$Hex$_1$NeuAc$_1$.

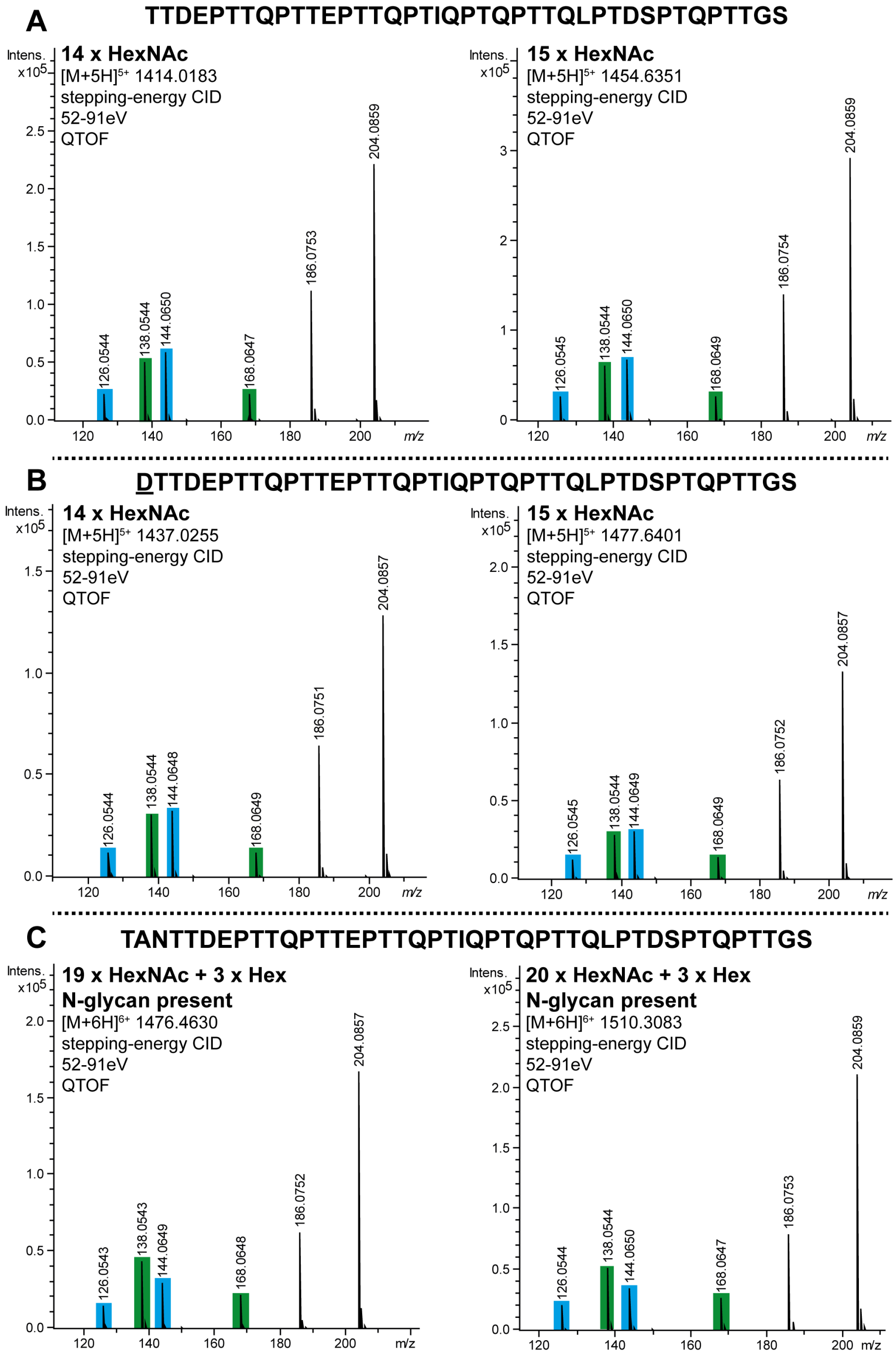

Figure S9: Zoom-in of the lower mass region HexNAc-related oxonium ions in stepping-energy CID of the heavily O-glycosylated region around Thr79-Ser121. A higher ratio of *m/z* 138 ($[HexNAc\text{-}CH_6O_3]^+$) + *m/z* 168 ($[HexNAc\text{-}2H_2O]^+$) compared to *m/z* 126 ($[HexNAc\text{-}C_2H_6O_3]^+$) + *m/z* 144 ($[HexNAc\text{-}C_2H_4O_2]^+$) are diagnostic for a GlcNAc-containing glycopeptide. More equal amounts of *m/z* 138 + *m/z* 168 compared to *m/z* 126 + *m/z* 144 are indicative for only GalNAc-containing glycopeptides [5]. (**A**) The glycopeptide $_{82}$TTDEPTTQPTTEPTTQPTIQPTQPTTQLPTDSPTQPTTGS$_{121}$ with $HexNAc_{14}$ and $HexNAc_{15}$ (**B**) The glycopeptide $_{81}$DTTDEPTTQPTTEPTTQPTIQPTQPTTQLPTDSPTQPTTGS$_{121}$ with $HexNAc_{14}$ and $HexNAc_{15.}$ (**C**) The glycopeptide $_{79}$TANTTDEPTTQPTTEPTTQPTIQPTQPTTQLPTDSPTQPTTGS$_{121}$ with $HexNAc_{19}Hex_3$ and $HexNAc_{20}Hex_{3.}$ Due to the large amount of GalNAc-containing O-glycans compared to the N-glycan portion in (**C**) the intensity of *m/z* 138 + *m/z* 168 compared to *m/z* 126 + *m/z* 144 is only slightly higher. In (**A**) and (**B**) *m/z* 138 + *m/z* 168 compared to *m/z* 126 + *m/z* 144 are almost equal indicating that the majority of the O-glycans are GalNAc-only-containing core 1 structures.

Released *N*-glycan analysis

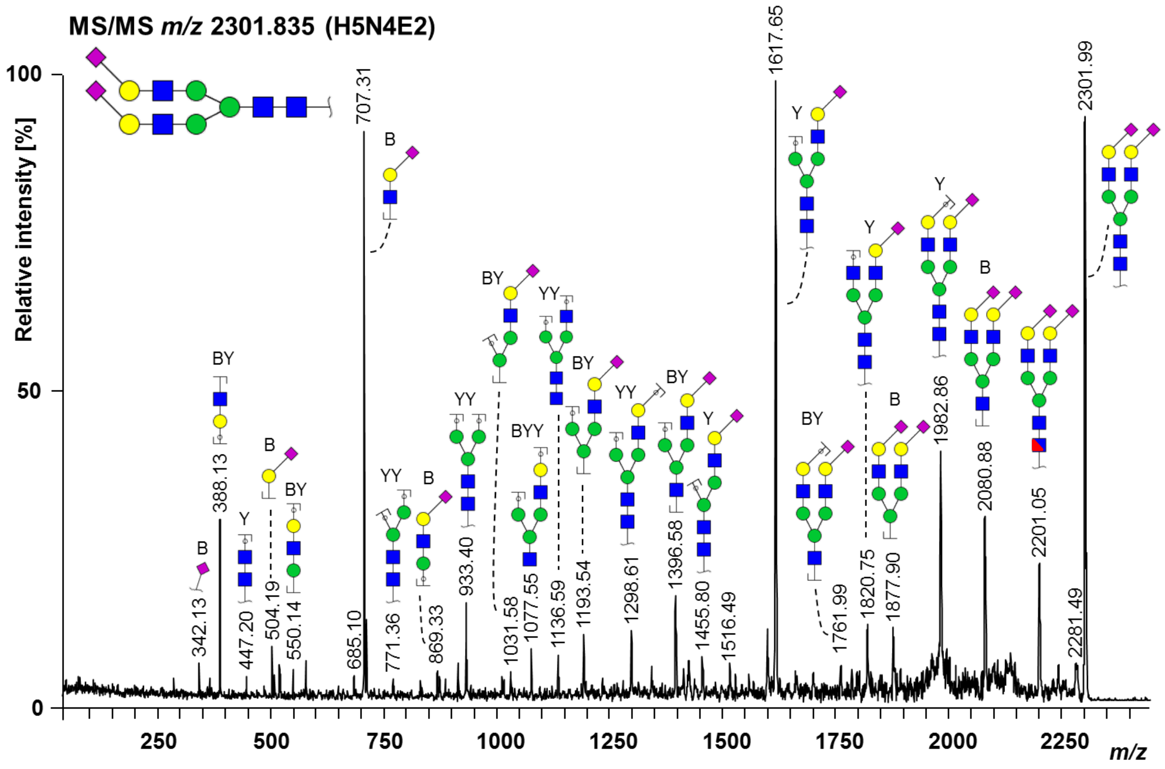

Figure S10. A representative MALDI-TOF/TOF-MS/MS spectrum containing the glycan structure $Hex_5HexNAc_4(\alpha2,6)NeuAc_2$ after linkage-specific sialic acid derivatization. Tandem MS/MS spectrum was obtained using fragmentation in gas-off TOF/TOF mode. Green circle = mannose; yellow circle = galactose; blue square = *N*-acetylglucosamine; red triangle = fucose; purple square with right angle= α2,6-linked *N*-acetylneuraminic acid.

Site-specific *N*-glycosylation site analysis

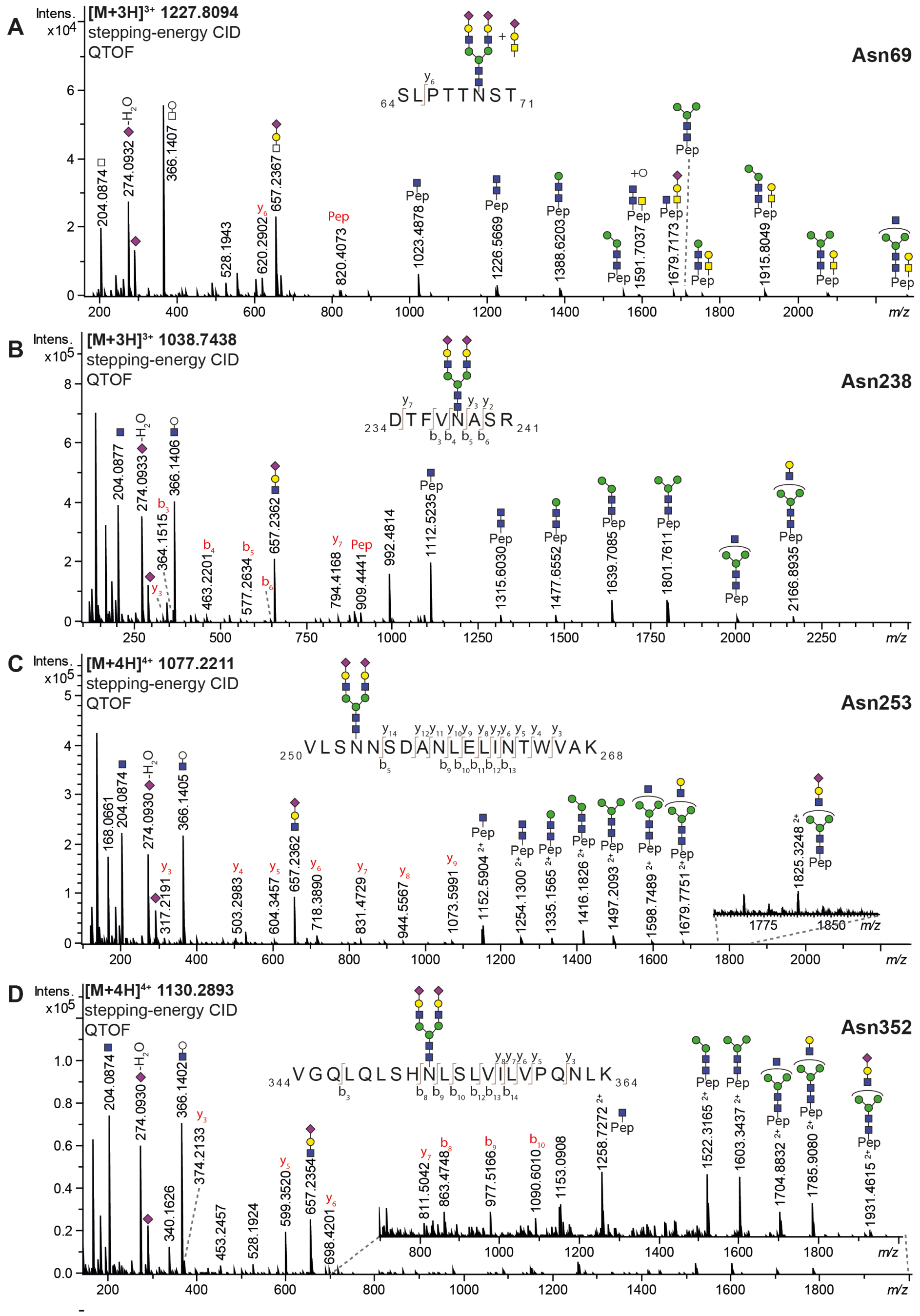

Figure S11: Representative stepping-energy fragmentation spectra confirming four of the six *N*-glycosylation sites of C1-Inh. MS/MS spectra are shown for (A) a Pronase-treated glycopeptide with the peptide sequence 64SLPTTNST71 carrying a monosialylated core 1 *O*-glycan and a diantennary disialylated *N*-glycan, confirming the *N*-glycosylation site Asn69; (B) a tryptic glycopeptide with the peptide sequence 234DTFVNASR241 with a diantennary disialylated *N*-glycan, confirming the *N*-glycosylation sites Asn238; (C) a tryptic glycopeptide with the peptide sequence 250VLSNNSDANLELINTWVAK268 with a diantennary disialylated *N*-glycan, confirming the *N*-glycosylation sites Asn253; (D) a tryptic glycopeptide with the peptide sequence 344VGQLQLSHNLSLVILVPQNLK364 with a diantennary disialylated *N*-glycan, confirming the *N*-glycosylation sites Asn352.

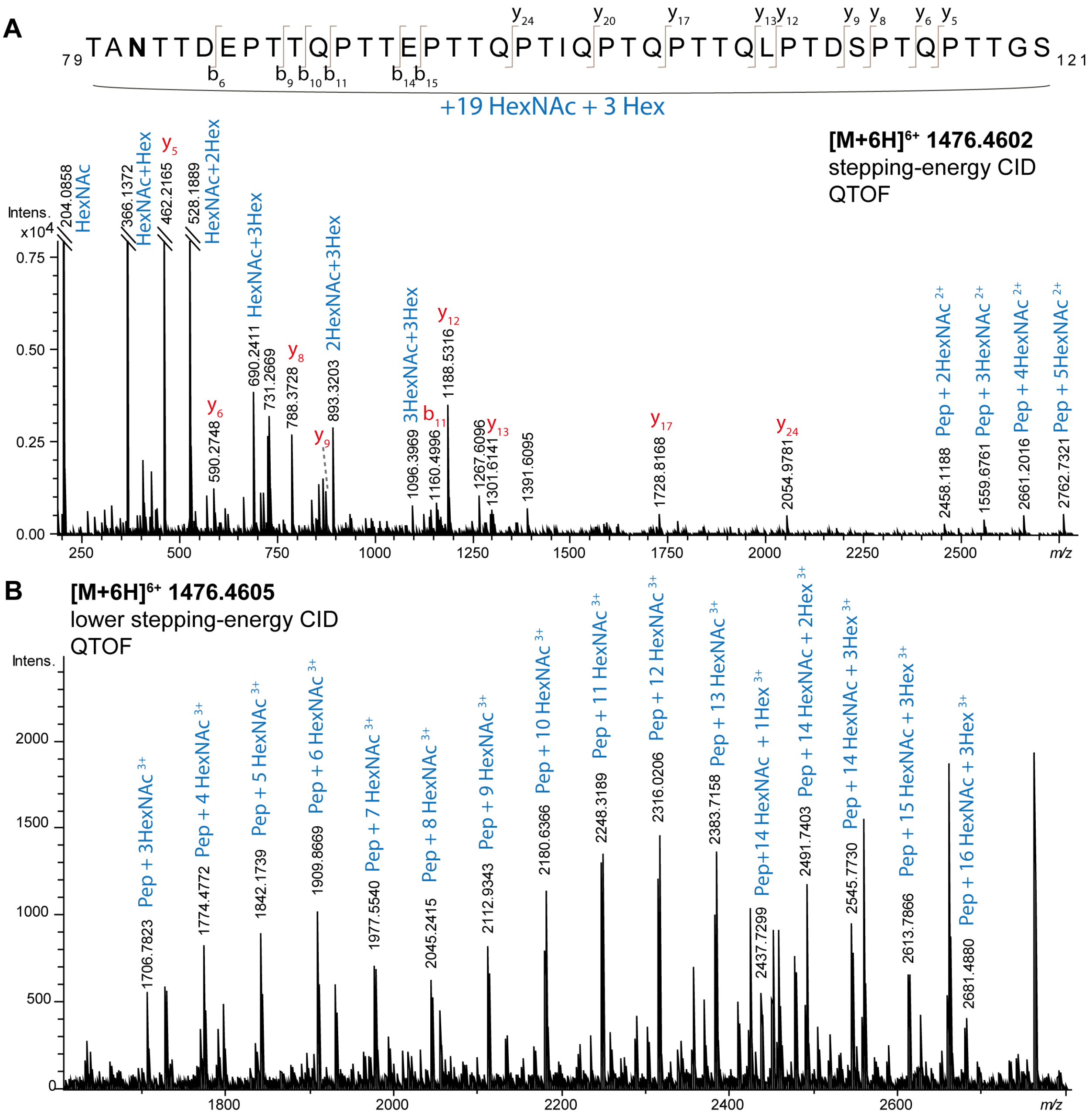

Figure S12: C18-PGC-LC-ESI−QTOF−MS/MS analysis of Pronase-generated *O*-glycopeptides with multiple glycosylation sites after PNGase F *N*-glycan release and exoglycosidase treatment with sialidase and galactosidase. (A) (B) Stepping-energy CID spectrum of the peptide 79TANTTDEPTTQPTTEPTTQPTIQPTQPTTQLPTDSPTQPTTGS121 with HexNAc19Hex3 attached. (B) Stepping-energy CID spectrum of the same glycopeptide as in panel A using lower stepping-energy CID (stepping energy is set to 60% and 80% each half of the time (instead of 80%-140) with a focus on the glycan-derived Y-ions. The spectra identify *N*-glycosylation site Asn81 due to diagnostic *N*-glycan oxonium ions such as HexNAc1Hex3, HexNAc2Hex3 and HexNAc3Hex3.

References

1. Plomp R, Hensbergen PJ, Rombouts Y, et al (2014) Site-specific N-glycosylation analysis of human immunoglobulin E. J Proteome Res 13:536–546.
2. Reiding KR, Blank D, Kuijper DM, et al (2014) High-throughput profiling of protein N-glycosylation by MALDI-TOF-MS employing linkage-specific sialic acid esterification. Anal Chem 86:5784–5793.
3. Selman MHJ, Hemayatkar M, Deelder AM, Wuhrer M (2011) Cotton HILIC SPE microtips for microscale purification and enrichment of glycans and glycopeptides. Anal Chem 83:2492–2499.
4. Jansen BC, Reiding KR, Bondt A, et al (2015) MassyTools: A High-Throughput Targeted Data Processing Tool for Relative Quantitation and Quality Control Developed for Glycomic and Glycoproteomic MALDI-MS. J Proteome Res 14:5088–5098.
5. Halim A, Westerlind U, Pett C, et al (2014) Assignment of saccharide identities through analysis of oxonium ion fragmentation profiles in LC-MS/MS of glycopeptides. J Proteome Res 13:6024–6032.
